# Vimentin intermediate filaments provide structural stability to the mammalian Golgi complex

**DOI:** 10.1242/jcs.260577

**Published:** 2023-10-18

**Authors:** Teresa Vitali, Rosa Sanchez-Alvarez, Tomasz M. Witkos, Ioannis Bantounas, Marie F. A. Cutiongco, Michal Dudek, Guanhua Yan, Alexander A. Mironov, Joe Swift, Martin Lowe

**Affiliations:** ^1^ School of Biological Sciences, Faculty of Biology, Medicine and Health, University of Manchester, The Michael Smith Building, Oxford Road, Manchester M13 9PT, UK; ^2^ Wellcome Centre for Cell-Matrix Research, University of Manchester, Manchester M13 9PT, UK; ^3^ Electron Microscopy Core Facility, Faculty of Biology, Medicine and Health, University of Manchester, The Michael Smith Building, Oxford Road, Manchester M13 9PT, UK

**Keywords:** Golgi complex, GORAB, Vimentin, Intermediate filament, Cell stiffness

## Abstract

The Golgi complex comprises a connected ribbon of stacked cisternal membranes localized to the perinuclear region in most vertebrate cells. The position and morphology of this organelle depends upon interactions with microtubules and the actin cytoskeleton. In contrast, we know relatively little about the relationship of the Golgi complex with intermediate filaments (IFs). In this study, we show that the Golgi is in close physical proximity to vimentin IFs in cultured mouse and human cells. We also show that the *trans*-Golgi network coiled-coil protein GORAB can physically associate with vimentin IFs. Loss of vimentin and/or GORAB had a modest effect upon Golgi structure at the steady state. The Golgi underwent more rapid disassembly upon chemical disruption with brefeldin A or nocodazole, and slower reassembly upon drug washout, in vimentin knockout cells. Moreover, loss of vimentin caused reduced Golgi ribbon integrity when cells were cultured on high-stiffness hydrogels, which was exacerbated by loss of GORAB. These results indicate that vimentin IFs contribute to the structural stability of the Golgi complex and suggest a role for GORAB in this process.

## Teaser

**Summary:** Vimentin intermediate filaments associate with the *trans*-Golgi network protein GORAB and provide structural stability to the Golgi complex in mammalian cells.

## INTRODUCTION

The Golgi complex has a characteristic structure in most eukaryotes, comprising a series of cisternae layered on top of each other to form the Golgi stack (Lowe, 2011; Shorter and Warren, 2002). In most vertebrate cells, the stacks are connected laterally to form the Golgi ribbon, which is usually positioned next to the centrosome by virtue of attachment to microtubules and the minus-end-directed microtubule motor protein dynein (Brownhill et al., 2009; Mascanzoni et al., 2022; Thyberg and Moskalewski, 1999). The Golgi is a polarized structure, comprising an entry (*cis*-) face for cargo entering from the endoplasmic reticulum (ER) (Brandizzi and Barlowe, 2013) and an exit (*trans*-) face from where cargo is transported to various downstream destinations (Di Martino et al., 2019). Cargo transiting the Golgi is exposed to a series of resident cisternal enzymes that sequentially modify the cargo, most notably at the level of glycosylation, as it moves from one side to the other (Glick and Nakano, 2009; Stanley, 2011; Welch and Munro, 2019). In addition to microtubules, the Golgi is also intimately associated with the actin cytoskeleton, which, in vertebrate cells, is not important for ribbon positioning (Egea et al., 2013). Instead, it functions at a more local level, contributing to formation of transport vesicles and linking of stacks into the Golgi ribbon (Chakrabarti et al., 2021). Actin contributes to the mechanical rigidity of Golgi membranes, which is important for the membrane deformation events that occur during transport vesicle formation (Guet et al., 2014). Actin can also couple to microtubules through the formin protein FHDC1 (or INF1), which is thought to coordinate actin and microtubule dynamics during Golgi ribbon formation (Copeland et al., 2016).

In contrast to microtubules and actin, much less is known about the association of the Golgi complex with the third major class of cytoskeletal elements, the intermediate filaments (IFs). IFs are present in all metazoans and consist of different proteins, depending on cell type and subcellular location (Etienne-Manneville, 2018; Herrmann et al., 2009). They provide important structural integrity to cells and contribute to a number of dynamic cell processes including cell migration, cell division and apoptosis (Etienne-Manneville, 2018; Herrmann et al., 2009). IFs are important for providing mechanical integrity to a number of cellular organelles, most strikingly in the case of the nucleus, where nuclear lamins play a key role (Gruenbaum and Foisner, 2015). Cytoplasmic vimentin IFs also contribute to the nuclear integrity by forming a cage around the nucleus that is mechanically protective (Patteson et al., 2019). A recent study has revealed that vimentin IFs can also associate with the ER and help maintain its morphological organization, as well as concentrate endolysosomes in the perinuclear area (Cremer et al., 2023). Vimentin cages can form around aggresomes to control protein degradation (Morrow and Moore, 2020; Morrow et al., 2020), and can also form around lipid droplets (Franke et al., 1987) and melanophores (Chang et al., 2009), and vimentin IFs also contribute to mitochondrial position and shape (Schwarz and Leube, 2016). In terms of the Golgi complex, previous work has shown that vimentin IFs can bind to Golgi membranes through the *cis*-Golgi-localised peripheral protein forminotransferase cyclodeaminase (FTCD, also known as 58K) (Gao and Sztul, 2001; Gao et al., 2002). However, the functional significance of this association remains unclear. It is also unknown whether the Golgi can physically associate with IFs in other ways, and any functional relationship between IFs and the Golgi complex remains poorly defined.

The coiled-coil Golgi protein GORAB, which is mutated in the skin and bone disorder gerodermia osteodysplastica (GO) (Hennies et al., 2008), forms discrete regions or domains at the *trans*-Golgi network (TGN) (Witkos et al., 2019). GORAB associates through Scyl1 and ARF1 with the COPI vesicle coat complex and has been proposed to function as a scaffold for COPI vesicle formation at the TGN (Witkos et al., 2019). GORAB is also present at the centrioles, where it binds the cartwheel protein SAS-6 (Kovacs et al., 2018). GORAB binds SAS-6 as a monomer, whereas at the TGN, it is present as a dimer (Fatalska et al., 2021). The function of GORAB at the centriole is unclear, but it might act as a physical scaffold, analogous to its proposed function at the TGN. In this study, we report that GORAB can physically associate with vimentin IFs and examine the relationship between vimentin IFs and Golgi architecture. Our results indicate that vimentin IFs are important to stabilize Golgi structure in mammalian cells, which is evident upon chemical perturbation of Golgi dynamics or culture in high mechanical stiffness. In contrast, GORAB does not play a major role in Golgi integrity, although it appears to sensitize the Golgi to the loss of vimentin IFs. These observations reveal a previously unappreciated role for vimentin IFs in providing structural stability to the Golgi complex.

## RESULTS

### GORAB can physically associate with vimentin IFs

We have previously shown that GORAB is present at the TGN in discrete membrane domains and that it can scaffold COPI assembly for retrograde vesicle transport (Witkos et al., 2019). However, the mechanisms underlying GORAB function remain poorly defined, and it is also unclear whether GORAB has additional functions at the TGN. To gain more insight into possible GORAB functions, we performed proximity biotinylation (BioID) using GORAB fused to the biotin ligase BirA to identify closely associated proteins (Roux et al., 2012). GORAB–BirA was stably expressed in HeLaM cells, where it correctly localized to the Golgi (Fig. S1A). Western blotting indicated that GORAB–BirA expression in the HeLaM cells was only modestly increased over that of the endogenous protein (twofold) (Fig. S1B). GORAB–BirA resulted in biotinylation of Golgi-associated proteins, as revealed by immunofluorescence microscopy (Fig. S1C) and western blotting (Fig. S1D). GORAB–BirA was also stably expressed in dermal fibroblasts, where it again localized correctly and led to biotinylation of Golgi proteins (Fig. S1E–G). Mass spectrometry (MS) of biotinylated proteins from both cell types identified a number of proteins, including GORAB itself, the COPI subunit γ2-COP (or COPG2) and Scyl1 (Tables S1 and S2), consistent with our earlier work on GORAB and COPI at the TGN and a previous study on *Drosophila* GORAB (Kovacs et al., 2018; Witkos et al., 2019). A number of other Golgi-associated proteins were also identified, together with various cytoskeletal proteins and proteins previously identified in other BioID screens, which are likely non-specific (Roux et al., 2012). Vimentin, although often found as a contaminant in proteomics experiments (https://reprint-apms.org/), was one of the most abundant biotinylated proteins in both cell types, prompting further investigation.

**Fig. 1.**
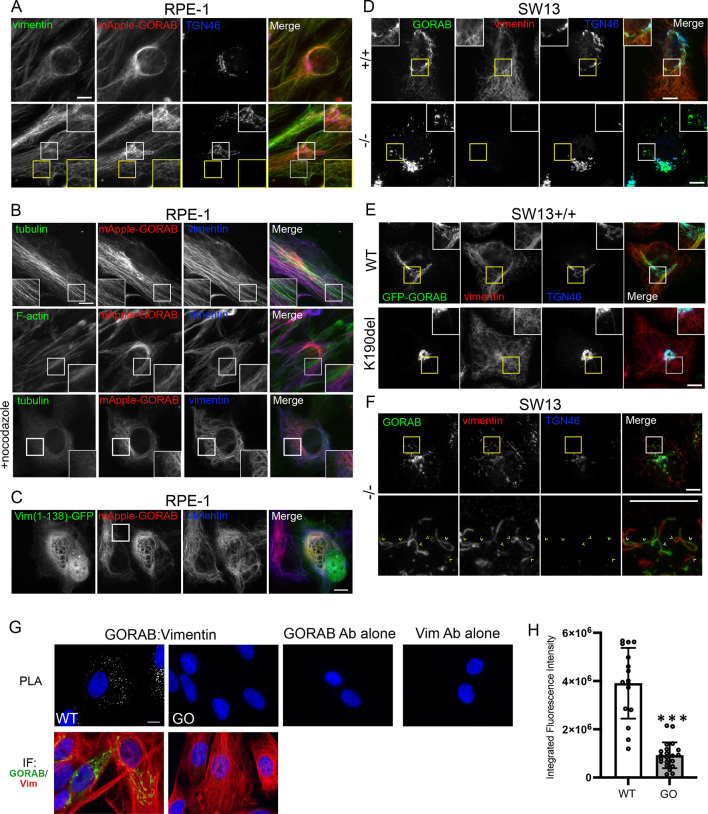
**Coalignment of overexpressed GORAB with vimentin intermediate filaments and endogenous GORAB–vimentin association**. (A) Immunofluorescence of stably transduced RPE-1 cells overexpressing mApple–GORAB and labelled with antibodies to vimentin and TGN46. (B) Immunofluorescence of RPE-1 cells overexpressing mApple–GORAB and labelled for microtubules (tubulin) and actin filaments (F-actin). The bottom row shows immunofluorescence of RPE-1 cells overexpressing mApple–GORAB treated with nocodazole for 2 h prior to fixation. (C) Immunofluorescence of RPE-1 cells overexpressing mApple–GORAB that were transiently transfected with a vector encoding GFP-tagged hamster vimentin (Vim)^1–138^. (D) Immunofluorescence of overexpressed untagged GORAB and vimentin filaments in SW13^+/+^ or SW13^−/−^ cells. (E) Immunofluorescence of SW13^+/+^ cells transduced with lentivirus expressing GFP-tagged wild-type (WT) and K190del mutant GORAB. (F) Immunofluorescence of SW13^−/−^ cells transduced with lentivirus expressing untagged GORAB. Cells with residual lower expression of vimentin are shown. Arrowheads indicate vimentin and GORAB colocalisation in fragmented filaments. (G) Top row: proximity ligation assay (PLA) performed on WT human dermal fibroblasts or dermal fibroblasts from an individual with GO, with primary antibodies to both proteins present (left two panels) or lacking primary antibody to either protein (right two panels). Bottom row: immunolabelling of the WT and GO fibroblasts with antibodies to GORAB (green) and vimentin (red). Images shown are representative of three independent experiments. All scale bars: 10 μm. (H) Quantification of the PLA signal in WT and GO fibroblasts. Error bars show s.d. Statistical significance was calculated using an unpaired two-tailed *t*-test. *n*=21. ***P<0.001.

To explore a possible physical association of GORAB with vimentin, GORAB was stably overexpressed in RPE-1 cells and colocalization with vimentin performed. As shown in Fig. 1A,B, when expressed at high levels, GORAB was able to form extensive filaments extending throughout the cytoplasm, which co-aligned with vimentin IFs. A similar observation was reported previously upon GORAB overexpression in HeLa cells and fibroblasts (Egerer et al., 2015). The GORAB filaments did not colocalize with microtubules or actin filaments (Fig. 1B). The co-alignment of GORAB with vimentin IFs was preserved following nocodazole treatment (Fig. 1B), which retracts the IF network as reported previously (Yoon et al., 1998), as well as after collapse of IFs by expression of a vimentin (Vim)^1–138^ dominant-negative mutant (Fig. 1C) that impairs IF assembly (Kural et al., 2007). GORAB fails to form filaments in SW13^−/−^ cells that lack all cytoplasmic IFs (Hedberg and Chen, 1986), instead forming ‘squiggles’, short filamentous elements reminiscent of structures seen during cytoplasmic IF assembly (Fig. 1D). Thus, the formation of extended GORAB filaments is dependent upon the presence of pre-existing vimentin IFs. The formation of GORAB filaments also requires GORAB oligomerisation, as the previously described K190 deletion (K190del) mutant, which cannot self-associate (Witkos et al., 2019) and is deficient in oligomerisation, even in the presence of an intact vimentin IF network (Fig. 1E). These results suggest that vimentin IFs can scaffold assembly of GORAB filaments, consistent with a physical interaction between the proteins, at least under the conditions of these experiments. Imaging of SW13^−/−^ cells with residual vimentin expression supported this conclusion, with GORAB apparently incorporated into or closely aligned with the fragmented IFs seen in these cells (Fig. 1F). It is worth noting that overexpression or loss of GORAB did not alter the vimentin IF network in wild-type (WT) cells (Fig. 1A,B; Fig. S2A,B), suggesting that GORAB does not play a role in the organization of IFs.

To investigate whether GORAB can associate with vimentin at endogenous levels, we performed an *in situ* proximity ligation assay (PLA) using antibodies against the proteins. The experiment was performed in human dermal fibroblasts, with GORAB-deficient fibroblasts from an individual with GO (hereafter, GO cells) used as a negative control, alongside controls in WT fibroblasts in which either the primary or secondary antibodies were omitted. As shown in Fig. 1G,H, a strong proximity ligation signal, indicated by fluorescent puncta, was observed in the WT cells, which was greatly diminished in the GO cells lacking GORAB, and no signal was detected in the antibody controls. These results indicate close proximity of GORAB and vimentin at endogenous levels, consistent with the proteins interacting. Taken together, our results indicate that GORAB has the ability to physically associate with vimentin IFs.

### Physical proximity of the Golgi complex to vimentin IFs

**Fig. 2.**
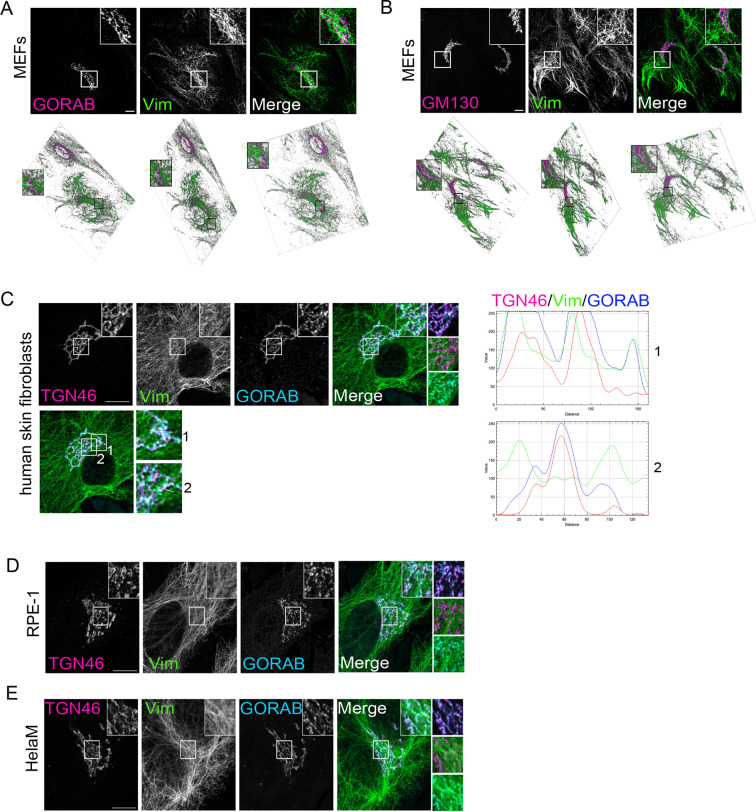
**Spatial relationship of GORAB, GM130, TGN46 and vimentin filaments in mouse and human cells.** (A,B) 2D (*z*-stack maximal-intensity projection) and 3D confocal images of WT MEFs stained with antibodies to vimentin (Vim) and either GORAB (A) or GM130 (B). (C–E) Deconvolved confocal *z*-stack maximal-intensity projections of human skin fibroblasts (C), RPE-1 (D) or HelaM (E) cells stained with antibodies against GORAB, TGN46 and vimentin. The white lines in C depict the regions used for calculating RGB fluorescence intensity profile plots in human skin fibroblasts, shown on the right. Images are representative of three independent experiments. All scale bars: 10 µm.

To better understand the spatial relationship of GORAB, and the Golgi complex more generally, with vimentin IFs, confocal microscopy was performed. As expected, vimentin IFs were observed to form a network extending throughout the cytoplasm, with an apparent enrichment in the perinuclear region where the Golgi is usually localised (Fig. 2). Confocal imaging of mouse embryonic fibroblasts (MEFs) revealed that both GORAB and the *cis*-Golgi marker GM130 (or GOLGA2) are not only in close physical proximity to the IF filaments, but apparently embedded in a meshwork of filaments, as observed in various tilted 3D projections (Fig. 2A,B). Further analysis was performed on human skin fibroblasts using confocal microscopy, followed by deconvolution to enhance the signal/noise ratio and better discern the spatial relationship of the TGN with vimentin IFs. This revealed that the TGN is also closely associated with vimentin IFs (Fig. 2C). The TGN occasionally could be seen to colocalise with IFs but, more frequently, it was aligned with IFs, with the IFs juxtaposed on one or both sides of the TGN membrane (Fig. 2C). GORAB puncta could be seen along the TGN, adjacent to the IFs. Similar results were obtained in RPE-1 and HeLa cells, where again the TGN membrane and GORAB were found colocalised with or juxtaposed on one or both sides by IFs (Fig. 2D,E). Because other cytoskeletal elements, such as microtubules, are also enriched in the perinuclear region of the cell and IFs associate with both microtubules and actin filaments, these observations do not necessarily show that the Golgi is physically associated with vimentin IFs. Nevertheless, they reveal an intimate spatial relationship between them.

### Loss of GORAB or vimentin has a modest effect on Golgi architecture at the steady state

**Fig. 3.**
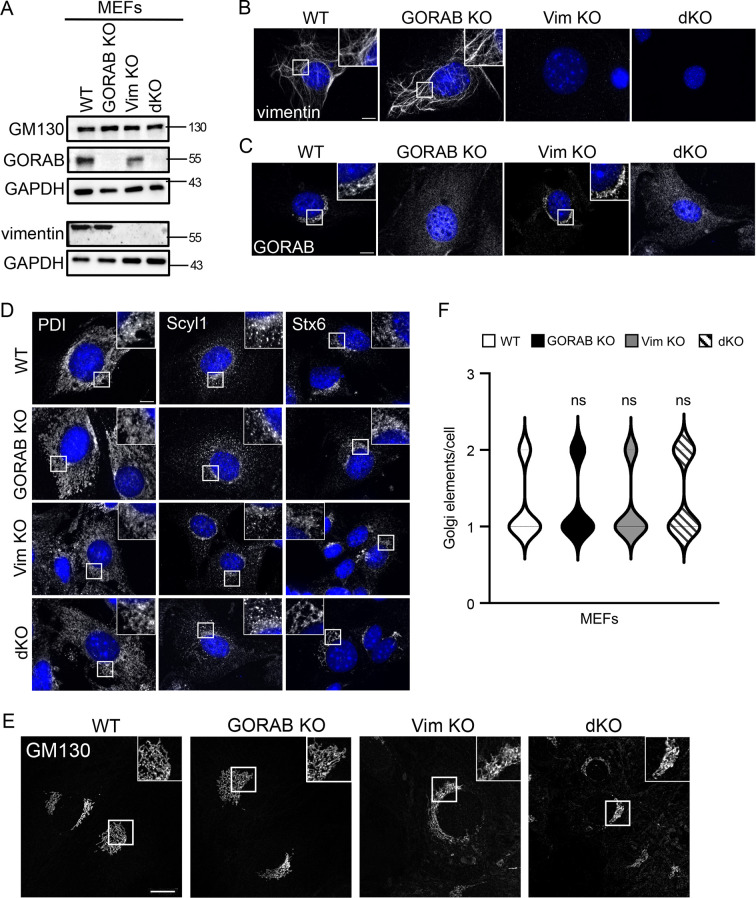
**Generation of GORAB KO in WT and Vim KO MEFs and effects upon ER and Golgi morphology.** (A) Western blot analysis of GORAB, vimentin and GM130 expression levels in WT, GORAB KO, Vim KO and double KO (dKO) MEFs. GAPDH was used as a loading control. (B,C) Immunofluorescence of vimentin (B) and GORAB (C) in WT, GORAB KO, Vim KO and dKO MEFs. (D) Immunofluorescence of the ER (PDI), ERGIC (Scyl1) and TGN (Stx6) in WT, GORAB KO, Vim KO and dKO MEFs. (E) Confocal images (*z*-stack maximal-intensity projection) of GM130 fluorescence in WT, GORAB KO, Vim KO and dKO MEFs. Images are representative of three independent experiments. All scale bars: 10 µm. (F) Analysis of the numbers of *cis*-Golgi (GM130) elements in WT, GORAB KO, Vim KO and dKO MEFs. In each case, comparisons are shown for the specified KO cell line versus WT cells, with statistical significance calculated using a non-parametric one-way ANOVA (Kruskal–Wallis test). *n*=30. ns, not significant.

**Fig. 4.**
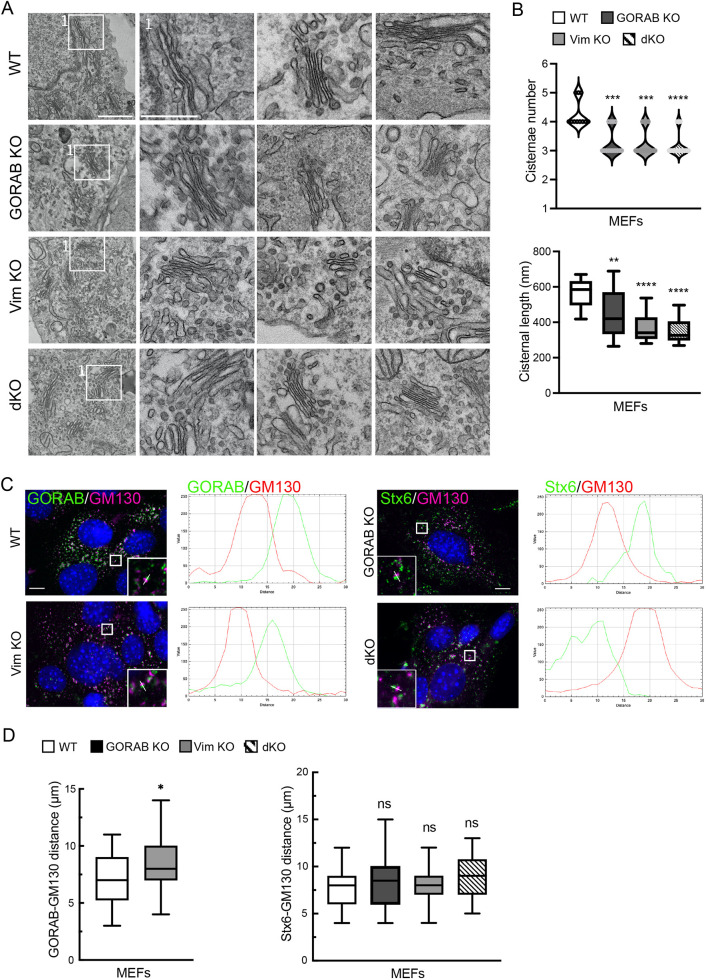
**Ultrastructure and polarisation of the Golgi in WT, GORAB KO, Vim KO and dKO MEFs.** (A) TEM images of the Golgi in WT, GORAB KO, Vim KO and dKO MEFs. The second column shows a magnified view of the inset box labelled in the left hand column (labelled 1 in each case). The other two columns show same magnification views of Golgi stacks from other KO cells (as indicated by row). Scale bars: 500 nm (left column); 250 nm (right columns). (B) Analysis of cisternae number and length from TEM images in WT, GORAB KO, Vim KO and dKO MEFs. In each case, comparisons are shown for the specified KO cell line versus WT cells, with statistical significance calculated using a non-parametric one-way ANOVA (Kruskal–Wallis test) (top) or one-way ANOVA with post hoc Dunnett test (bottom). *n*=40. (C) Immunofluorescence of *cis*-Golgi (GM130) and TGN (GORAB or Stx6) polarization in WT, GORAB KO, Vim KO and dKO MEFs 2 h after nocodazole treatment. White lines depict the regions used for calculating RGB fluorescence intensity profile plots. Scale bars: 10 µm. (D) Analysis of *cis*- and TGN Golgi stack distance in WT, GORAB KO, Vim KO and dKO MEFs. In each case, comparisons are shown for the specified KO cell line versus WT cells, with statistical significance calculated using an unpaired two-tailed *t*-test (left) or one-way ANOVA with post hoc Dunnett test. *n*=40. For box plots in B,D, boxes show the 25–75th percentiles, whiskers show the default Tukey values calculated in GraphPad Prism, and the median is marked with a line. ns, not significant; *P≤0.05; **P<0.01; ***P<0.001; ****P<0.0001.

We next tested whether vimentin IFs play a role in Golgi integrity, positioning or morphology. In parallel, we investigated whether GORAB might also play a role in these processes, independently or in conjunction with vimentin. For this purpose, we used vimentin-null MEFs that were obtained from a previously generated vimentin knockout (KO) mouse (Colucci-Guyon et al., 1994), and performed CRISPR/Cas9-mediated KO of GORAB in these cells to make a double KO or, in WT MEFs, a single-KO cell line. Western blotting and immunofluorescence microscopy confirmed the knockout of vimentin and GORAB in the single and double KO lines, as expected (Fig. 3A–C). There was no major change in the morphology or positioning of the ER (labelled using PDI, also known as P4HB), the ER to Golgi intermediate compartment (ERGIC, labelled using Scyl1), or the Golgi complex [labelled using the TGN marker syntaxin 6 (Stx6)] in the single and double KO cells (Fig. 3D). Confocal imaging of the *cis*-Golgi marker GM130 indicated that the Golgi ribbon was intact in all KO lines (Fig. 3E,F). Transmission electron microscopy (TEM) was next used to examine Golgi ultrastructure in the KO cells. This revealed a slight reduction in cisternal number and a shortening of the cisternal length in all three KO lines (Fig. 4A,B). There also appeared to be more circular profiles in the KO cells, which might correspond to vesicles or tubules. Consistent with the presence of stacked cisternae in the KO cells, *cis–trans* polarity of the Golgi, assessed by immunofluorescence microscopy of nocodazole-induced Golgi ministacks, was maintained (Fig. 4C,D). Golgi polarity was also maintained in the SW13^−/−^ cells lacking IFs (Fig. S2C). Thus, although there were changes in Golgi morphology in the KO cells, these were fairly modest, and ribbon integrity appeared unaffected. Taken together, these results indicate that GORAB and vimentin play only a modest role in Golgi organization under steady-state conditions.

### Loss of vimentin causes faster disassembly and slower reassembly of the Golgi complex upon chemical perturbation

**Fig. 5.**
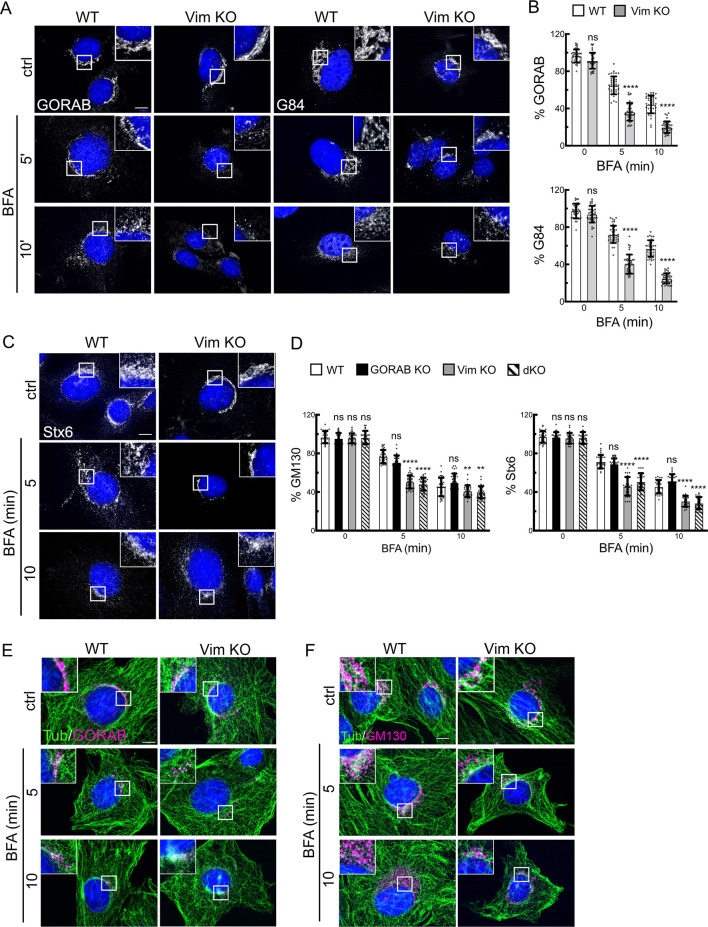
**Golgi fragmentation in WT and Vim KO MEFs treated with BFA.** Cells were incubated in the presence of BFA (5 µg/ml) at 37°C for the indicated times prior to fixation and immunostaining. (A,C) Immunofluorescence of GORAB and Golgin-84 (G84) (A) and Stx6 (C) in WT and Vim KO MEFs. (B,D) Quantification of GORAB and Golgin-84 (B) and GM130 and Stx6 (F) fluorescence intensities in WT, GORAB KO, Vim KO and dKO MEFs treated for 5 and 10 min with BFA. At each timepoint, comparisons are shown for the specified KO cell line versus WT cells. Statistical significance between WT and KO cells was calculated using an unpaired two-tailed *t*-test (B) or two-way ANOVA with post hoc Dunnett test (D). Error bars show s.e.m. *n*=40. (E) Immunofluorescence of GORAB with tubulin in WT and Vim KO MEFs. (F) Immunofluorescence of GM130 with tubulin in WT and Vim KO MEFs. Scale bars: 10 µm. ns, not significant; ****P<0.0001.

To further explore whether vimentin IFs play a structural role at the Golgi, we used the drug brefeldin A (BFA) to trigger Golgi disassembly in control or KO MEFs. BFA causes disassembly of the Golgi complex by inhibiting ARF guanine nucleotide exchange activity, which can be reversed by washing out the drug (Klausner et al., 1992). We reasoned that if vimentin IFs (or GORAB) provide underlying mechanical support to the Golgi, then their loss would result in faster Golgi fragmentation upon BFA treatment. For these experiments, we focused on the initial stages of Golgi disassembly, occurring over 10 min. As shown in Fig. 5A,C and Fig. S3A,B, as expected, treatment of WT MEFs with BFA caused disassembly of the Golgi, visualized using antibodies against GORAB and markers for the *cis*- [Golgin-84 (or GOLGA5) and GM130] and *trans*- (Stx6) Golgi. Disassembly was quantified by measuring the immunofluorescence signal above threshold, which confirmed the progressive loss of Golgi signal over the first 10 min of incubation (Fig. 5B,D). Loss of vimentin accelerated disassembly of the Golgi complex, as indicated by all Golgi markers (Fig. 5A–D). A similar result was seen with the double KO cells, whereas loss of GORAB alone did not affect the rate of disassembly (Fig. 5D; Fig. S3A,B). To further assess the kinetics of Golgi disassembly, live imaging was performed in WT and vimentin KO cells expressing the Golgi marker NAGT1 (or MGAT1) fused to GFP (referred to as NAGFP) (Shima et al., 1997). This confirmed the faster Golgi disassembly in vimentin KO cells (Fig. S3C,D; Movies 1 and 2) and, strikingly, indicated increased tubulation of the Golgi membranes at early timepoints during the disassembly process (Fig. S3E; Movies 3 and 4). This is consistent with reduced mechanical support of the membranes in the absence of vimentin. To further assess the role of IFs in Golgi stability, the experiment was repeated in SW13^−/−^ cells lacking all cytoplasmic IFs. As seen in the MEFs, there was a more rapid fragmentation of the Golgi complex upon BFA treatment in the SW13^−/−^ cells compared to that in WT SW13^+/+^ cells (Fig. S4A,B). The microtubule network appeared normal in the vimentin KO MEFs (Fig. 5E,F), and this was also the case in SW13^−/−^ cells (Fig. S4C), indicating that the changes in Golgi stability and GORAB localization are not due to indirect effects on the microtubule cytoskeleton. Similarly, we did not see any changes in filamentous actin organization in the vimentin or double KO MEFs or SW13^−/−^ cells, or in focal adhesions, again arguing against indirect effects via the actin cytoskeleton when vimentin IFs were missing (Fig. S5A,B).

We next used nocodazole, which causes microtubule disassembly, as another way to induce Golgi disassembly. Upon nocodazole treatment, the Golgi ribbon is converted to mini-stacks that localise adjacent to ER exit sites (Cole et al., 1996). Nocodazole induced loss of Golgi ribbon integrity in WT cells, and this effect was accelerated in vimentin KO cells (Fig. S6A,B). Thus, loss of vimentin caused a faster disassembly of the Golgi complex upon microtubule depolymerisation, consistent with reduced structural support for the Golgi in its absence. This result also suggests that effects on Golgi stability are independent of indirect effects via microtubules, which can also associate with vimentin IFs (Schaedel et al., 2021).

**Fig. 6.**
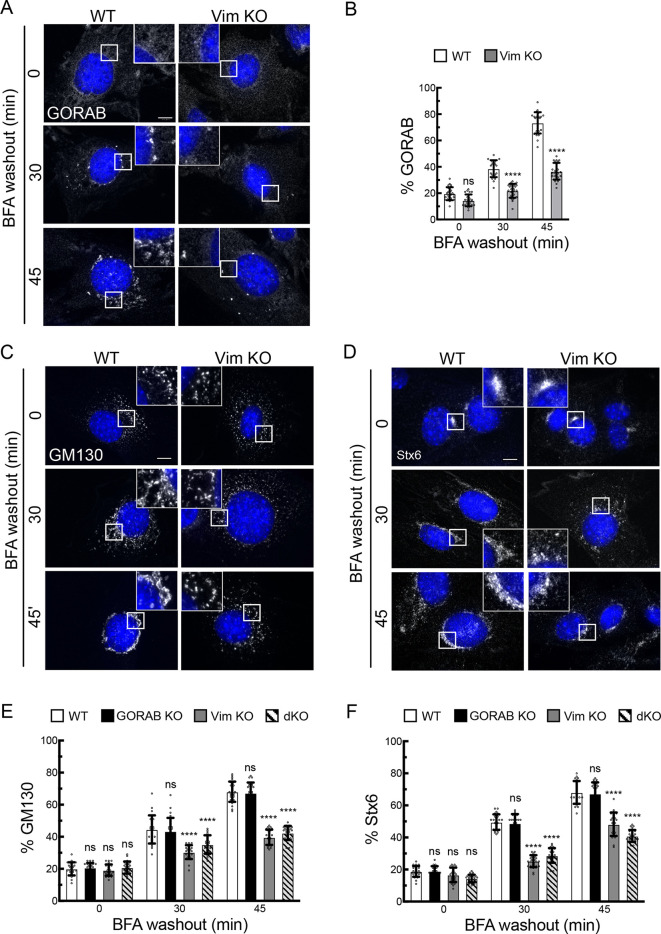
**Golgi reassembly in WT and Vim KO MEFs upon BFA washout.** Cells were incubated with BFA (5 µg/ml) for 90 min at 37°C, washed and incubated in fresh medium for the indicated time points, prior to fixation and immunostaining. (A) Immunofluorescence of GORAB in WT and Vim KO MEFs after BFA washout. (B) Quantification of GORAB reassembly in WT and Vim KO MEFs. Comparison between groups was performed with an unpaired two-tailed *t*-test. *n*=30. (C,D) Immunofluorescence of GM130 (C) and Stx6 (D) in WT and Vim KO MEFs after BFA washout. (E,F) Quantification of GM130 (E) and Stx6 (D) reassembly in WT, GORAB KO, Vim KO and dKO MEFs. At each timepoint, comparisons are shown for the specified KO cell line versus WT cells, with statistical significance calculated using a two-way ANOVA with post hoc Dunnett test. *n*=30. All scale bars: 10 µm. Error bars show s.e.m. ns, not significant; ****P<0.0001.

To further assess a possible structural role for vimentin IFs in Golgi organization, the rate of Golgi reassembly following BFA washout was analyzed. The MEFs were treated with BFA for 90 min to induce complete Golgi fragmentation, which was seen in WT and all three KO cell lines (Fig. 6A,C,D, 0 min washout). GM130 was present in puncta corresponding to ER exit sites as reported previously (Mardones et al., 2006), whereas Stx6 was concentrated at the centrosome, in line with previous studies showing TGN concentration at the centrosome upon BFA treatment of rodent cells (Reaves and Banting, 1992). After 30 min washout of BFA, Golgi elements containing the markers GM130 and Stx6 started to concentrate in the perinuclear region of WT cells, consistent with the reassembly of the Golgi ribbon, which had further progressed at 45 min following washout (Fig. 6C,D). Interestingly, the appearance of GORAB in the reforming Golgi ribbon appeared slower than the other markers (Fig. 6A). Reassembly in the vimentin KO cells was significantly delayed compared to that in WT MEFs, as indicated by GORAB (Fig. 6A,B), GM130 (Fig. 6C,E) and Stx6 (Fig. 6D,F) staining. A similar result was obtained with the double KO cells, whereas loss of GORAB alone did not affect the kinetics of Golgi reassembly (Fig. 6E,F; Fig. S7A). Live imaging also indicated slower assembly and revealed tubulation of the Golgi membranes during reassembly in the vimentin KO cells, which was less apparent in WT cells (Fig. S7B; Movies 5 and 6). Although the mechanisms underlying this effect are unclear, it is consistent with a reduced ability of Golgi membranes to organize into a ribbon as reassembly proceeds in the absence of vimentin. Golgi reassembly was also delayed in SW13^−/−^ cells compared to that in WT SW13^+/+^ cells, confirming the effect was due to loss of IFs (Fig. S4E,F). The Golgi ultimately fully reformed at later timepoints in all cell lines, indicating that the loss of vimentin only slows, rather than arrests, Golgi reassembly under these conditions (Fig. S7C). Finally, Golgi reassembly upon washout of nocodazole was also slower in vimentin KO cells (Fig. S6C,D). Taken together, the results support a role for vimentin in scaffolding Golgi membranes such that they undergo faster disassembly and slower reassembly in its absence.

### Loss of vimentin destabilizes Golgi structure in a high-stiffness environment

**Fig. 7.**
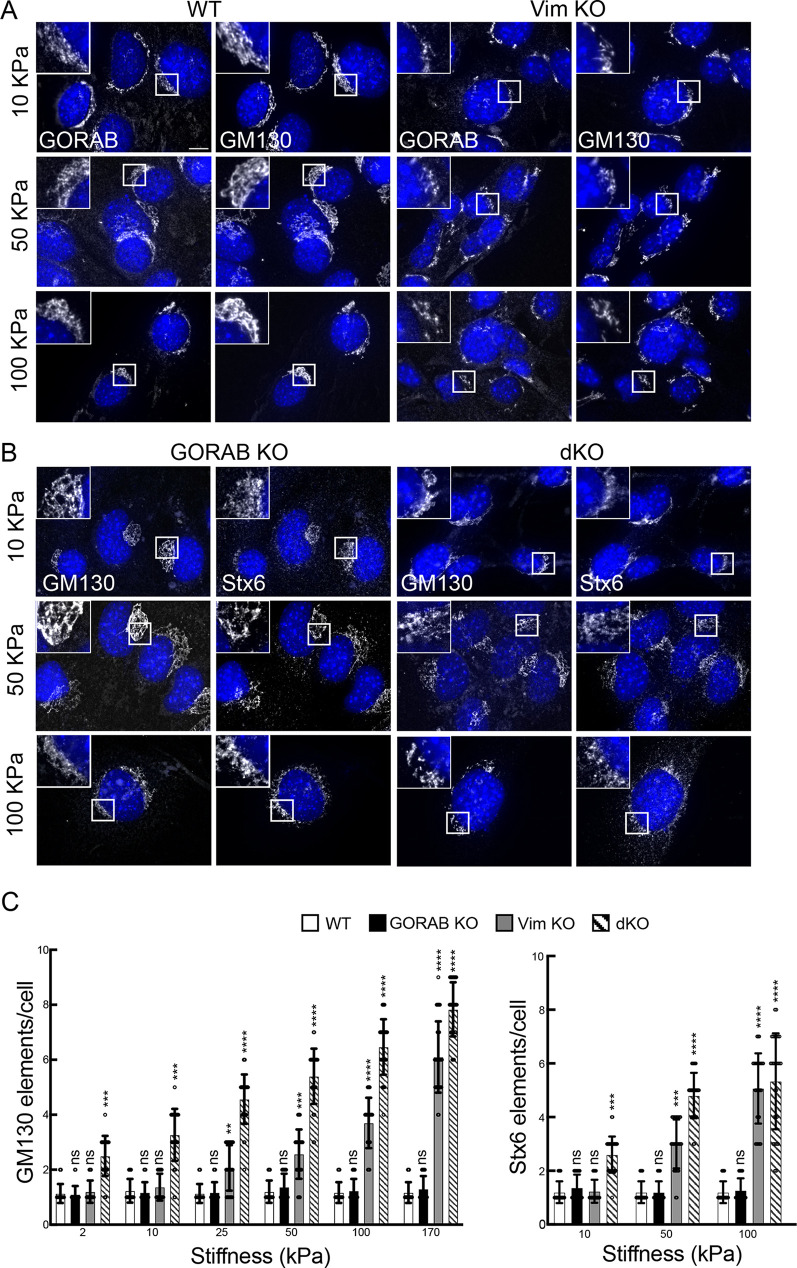
**Golgi morphology in the KO MEFs in response to different stiffness substrates.** The indicated cells were plated on elastic Matrigel-coated coverslips for 48 h prior to fixation and immunostaining. (A,B) Cells were labelled with antibodies to GORAB and GM130 (A), and GM130 and Stx6 (B). Scale bars: 10 µm. (C) Analysis of the numbers of *cis*-Golgi (GM130) and TGN (Stx6) elements in WT, GORAB KO, Vim KO and dKO MEFs upon culture in different stiffnesses. At each stiffness, comparisons are shown for the specified KO cell line versus WT cells, with statistical significance calculated using a non-parametric one-way ANOVA (Kruskal–Wallis test). Error bars show s.e.m. *n*=30. ns, not significant; ***P<0.001; ****P<0.0001.

The vimentin IF network provides mechanical support to cells, but the extent to which it can mechanically support individual organelles is less clear. To examine whether vimentin IFs might play a role in providing mechanical support to the Golgi complex, cells were cultured on hydrogel substrates of differing stiffness, ranging from 2 kPa (soft) to 170 kPa (stiff). In terms of physiological comparison, these would be of the order seen in soft tissues such as brain and adipose versus stiffer tissues such as precalcified bone and cartilage, respectively (Discher et al., 2009; Guimarães et al., 2020). In WT MEFs, the Golgi appeared unaffected in terms of ribbon morphology on the different substrates (Fig. 7A,C). This indicates that, at least in MEFs, the Golgi does not undergo significant changes in its organization when substrate stiffness is altered. A similar result was seen with GORAB KO MEFs (Fig. 7B,C). However, in vimentin KO MEFs, there was a progressive increase in fragmentation of the Golgi ribbon as substrate stiffness increased (Fig. 7A,C). The extent of fragmentation was greater in the double KO cells compared to vimentin-only KO cells, an effect that was particularly evident at lower stiffnesses (Fig. 7B,C). Analysis of microtubules and actin filaments in the vimentin KO cells indicated no gross changes in these cytoskeletal elements compared to those in controls when grown on different stiffness substrates, suggesting that increased Golgi fragmentation is independent of these elements (Fig. S5C,D). These observations suggest that vimentin IFs provide mechanical support to the Golgi ribbon, which is important in higher-stiffness environments, and that GORAB also contributes to the mechanical integrity of the Golgi ribbon.

## DISCUSSION

Although cytoplasmic IFs are known to provide mechanical support to cells, their functional relationship with organelles is less well understood. In particular, the extent to which IFs associate with the Golgi complex and the role of any association remain poorly defined. Our data indicate a close spatial relationship between the Golgi and vimentin IFs, and that loss of vimentin reduces the structural integrity of the Golgi ribbon. How vimentin IFs provide support to the Golgi remains to be determined, but an attractive hypothesis is that they form a cage or meshwork around the organelle, which provides a mechano-protective function. This would be analogous to what has been reported for the nucleus and other cytoplasmic organelles (Cremer et al., 2023; Etienne-Manneville, 2018). The Golgi ribbon, by virtue of its localization adjacent to the nucleus, might be embedded within the IF meshwork that forms a cage around the nucleus (Cremer et al., 2023; Etienne-Manneville, 2018). This would certainly be consistent with our imaging data. The integrity of the Golgi might therefore be controlled by the same network of vimentin IFs that maintain nuclear integrity and integrity of other perinuclear organelles, allowing for a coordinated control of organelle mechanics.

Although vimentin KO mice develop with no overt phenotype (Colucci-Guyon et al., 1994), numerous studies have shown a range of phenotypes in these mice that are evident at the cellular and tissue level, including wound healing defects, altered cell differentiation, and various vascular, renal and metabolic defects (Ridge et al., 2022). The extent to which disruption of Golgi integrity contributes to the described *in vivo* phenotypes remains to be determined. It is also currently unclear whether the changes in Golgi integrity we observed upon loss of vimentin translate to defective trafficking or cargo processing at the Golgi. The increase in vesicular profiles seen in electron microscopy, combined with delayed Golgi reassembly upon washout of BFA and nocodazole, suggest altered trafficking kinetics, but this will require further investigation.

Whether the Golgi binds directly to vimentin IFs and the possible role of any physical interaction remain poorly defined. Previous studies identified the Golgi-localised metabolic enzyme FTCD as a vimentin-binding protein (Gao and Sztul, 2001), but the significance of this interaction for Golgi integrity or function remains unclear. FTCD overexpression alters vimentin IF organization as opposed to affecting Golgi morphology per se, and it was proposed that the Golgi might act as a reservoir for FTCD that in turn might modulate vimentin dynamics (Gao and Sztul, 2001; Gao et al., 2002). In this study, we identified the TGN-localized coiled-coil protein GORAB as a possible vimentin interactor. Our PLA data support an interaction between the proteins at endogenous levels, and as previously reported for FTCD, GORAB associated with vimentin IFs when overexpressed in cells, although in contrast to FTCD, overexpressed GORAB did not affect the organization of IFs. Loss of GORAB did not affect Golgi ribbon morphology at the steady state, although in high-stiffness conditions, it did sensitize the Golgi to loss of vimentin. Whether GORAB binding to vimentin provides a direct physical link between the TGN membrane and vimentin IFs remains an open question. It is interesting to note that GORAB oligomerisation is necessary for binding to vimentin IFs and it is also required for the formation of TGN-associated GORAB domains involved in COPI vesicle trafficking (Witkos et al., 2019). Hence, GORAB oligomerisation might control the various functions of the protein, as proposed by Fatalska et al. (2021), who found that centriolar GORAB is present as a monomer, whereas it is a dimer at the TGN. Interestingly, the K190 deletion, a causative mutation in GO that disrupts GORAB oligomerisation, abolishes both TGN domain formation and IF binding, raising the possibility that IF binding could also be disrupted in GO. FTCD was previously shown to bind Scyl1 (Burman et al., 2010), which is a binding partner of GORAB (Di et al., 2003; Witkos et al., 2019), raising the additional possibility that GORAB might form a common complex with FTCD that can bind to vimentin IFs. It is also, of course, possible that the Golgi might bind to vimentin via other proteins, which remain to be identified.

Previous studies have shown that the Golgi complex can act in a mechanoresponsive manner, with changes in cell mechanics resulting in altered Golgi function (Guet et al., 2014; Romani et al., 2019). Using a micro-rheology approach, Guet et al. (2014) showed that Golgi membrane tension can influence the generation of Golgi-derived Rab6-positive vesicles. They also showed that Golgi membrane tension is dependent upon the actin cytoskeleton. Similarly, it was recently shown that ER–Golgi trafficking of the SREBP transcription factor, which controls lipid metabolism, is also mechanoresponsive, again downstream of the actin cytoskeleton (Romani et al., 2019). The actin cytoskeleton can therefore dictate the functional response of the Golgi complex to changes in cell mechanics. Our data suggest that vimentin IFs also play a role in this response. For example, we could hypothesize that by providing mechanical support to Golgi membranes, vimentin IFs could influence rates of vesicle formation at this organelle, a process which is dependent upon the extent of physical deformation of the Golgi membrane. Similarly, maintaining the integrity of the Golgi ribbon is likely to have important functional consequences, considering that an intact Golgi ribbon is required to maintain optimum protein glycosylation (Puthenveedu et al., 2006; Xiang et al., 2013), guide polarized secretion (Ravichandran et al., 2020; Yadav et al., 2009) and can influence the progression through the cell cycle (Mascanzoni et al., 2022; Rabouille and Kondylis, 2007; Sutterlin et al., 2002). It will be interesting to investigate the role of vimentin IFs in maintaining these and other aspects of Golgi function, as well as the extent of functional interplay between IFs and the actin cytoskeleton at the Golgi.

An apparent conundrum from our experiments is that loss of vimentin strongly affected the integrity of the Golgi ribbon in cells grown on stiff hydrogels, but appeared dispensable for cells growing at the steady state on plastic or glass, which are orders of magnitude stiffer than the highest stiffness gel we used (∼1 GPa versus 170 kPa). These results suggest that the nature of the substrate as well as its inherent stiffness are important for the responsiveness of the Golgi membranes to loss of vimentin. Glass and plastic lie far beyond the physiological range of stiffness found within tissues and they are also clearly chemically distinct to the extracellular matrix found within tissues and also to the hydrogels we used. Considering that vimentin IFs bind to focal adhesions and there is functional cross-talk between these structures (Kreis et al., 2005; Leube et al., 2015), it is perhaps not surprising that changes in dependencies upon vimentin loss will differ depending on the chemical nature of the cellular environment. In addition, it is known that changes in material chemistry can influence the conformation of fibronectin displayed to cells (Garcia et al., 1999; Rico et al., 2009), which could also contribute to the changes we observe between glass (with passively adsorbed fibronectin from the medium) and hydrogels (with covalently attached fibronectin). By using the same chemical substrate in our hydrogels, we were able to differentiate between effects due to cell substrate versus effects due to stiffness, and our results indicate a clear dependency upon vimentin for Golgi stability at high, but not low, stiffness. Clearly, it will be interesting to perform further studies using both 2D and 3D culture on substrates of different composition, assessing the role of vimentin in maintaining Golgi integrity under the different conditions.

## MATERIALS AND METHODS

### Antibodies

The following antibodies were used in this study: rabbit anti-GORAB [Proteintech, 17798-1-AP, 1:500 for western blotting (WB) and 1:100 for immunofluorescence (IF)]; rat anti-vimentin (R&D Systems, MAB21052, 1:500 for WB and 1:100 for IF); mouse anti-vimentin (Santa Cruz Biotechnology, V9, sc-6260, 1:100 for IF); mouse anti-vinculin (Sigma-Aldrich, V9131, 1:200 for IF); rabbit anti-GM130 (MLO7 anti-N73pep; Nakamura et al., 1997; 1:1000 for WB and 1:100 for IF); mouse anti-GM130 (BD Biosciences, 610822, 1:1000 for WB and 1:100 for IF); mouse anti-Stx6 (a gift from Andrew Peden, University of Sheffield, 1:50 for IF); sheep anti-TGN46 (a gift from Vas Ponnambalam, University of Leeds, 1:300 for IF); mouse anti-PDI (Enzo Life Sciences, ADI-SPA-891, 1:1000 for WB and 1:100 for IF); TAT-1 mouse anti-α-tubulin (a gift from Keith Gull, University of Oxford, 1:1000 for WB and 1:100 for IF); mouse anti-α-tubulin (Sigma-Aldrich, T6199, 1:100 for IF); mouse anti-GAPDH (Santa Cruz Biotechnology, sc-365062, 1:2000 for WB); mouse anti-Myc (Cell Signalling Technology, 9B11, 1:1000 for IF); and goat anti-lamin B (Santa Cruz Biotechnology, sc-6216, 1:100 for IF). Anti-Scyl1 antibodies were raised in sheep against recombinant GST-tagged full-length human Scyl1 and affinity purified against the recombinant protein. Antibodies were validated by WB and IF of Scyl1 KO cells. Sheep anti-Scyl1 was used at 1:500 for WB and 1:50 for IF. Actin was labelled with Alexa Fluor 594-conjugated phalloidin (Thermo Fisher Scientific) at 1:1000. HRP-conjugated secondary antibodies for immunoblotting were from Sigma-Aldrich (A4416 and A0545, both at 1:1000). Fluorophore-conjugated secondary antibodies for immunofluorescence were from Jackson ImmunoResearch laboratories [cat nos. 712-545-150, 705-545-003, 711-545-152, 715-545-150, at 1:200 dilution (Alexa Fluor 488 conjugates); cat nos. 712-585-150, 705-585-003, 711-585-152, 715-585-150, at 1:800 dilution (Alexa Fluor 594 conjugates); cat nos. 705-605-003, 711-605-152, 715-605-150 at 1:200 dilution (Alexa Fluor 647 conjugates)]. HRP- or Alexa Fluor 488-conjugated streptavidin for BioID experiments was from Thermo Fisher Scientific.

### Cell culture

HeLaM, hTERT-RPE-1 and WT MEF cells were from American Type Culture Collection (ATCC, Manassas, VA, USA). Vimentin KO MEFs from a vimentin^-^null mouse model were a kind gift from Dr Sara Koester (University of Gottingen) (Colucci-Guyon et al., 1994). Human skin fibroblasts derived from unaffected individuals and from individuals with GO were obtained from Dr May Tassabehji (University of Manchester, UK) and from the Cell Line and DNA Bank from Patients affected by Genetic Diseases (Genova, Italy). SW13 clone 1 (vim^+/+^) and clone 2 (vim^−/−^) were kind gifts from Victoria Allan (University of Manchester). All cells apart from hTERT-RPE-1 were cultured in GlutaMAX Dulbecco's modified Eagle medium (DMEM; Thermo Fisher Scientific, 31966021) supplemented with 10% fetal bovine serum (FBS; Thermo Fisher Scientific, cat. no. A5256801), 50 U/ml penicillin and 50 µg/ml streptomycin. hTERT-RPE-1 cells were cultured in Ham's F12 and DMEM 1:1 (Thermo Fisher Scientific, 11320033) with the same supplements. Cells were cultured at 37°C in 5% CO_2_. All cell lines were routinely tested for mycoplasma contamination.

### Growth of cells on polyacrylamide hydrogels

Polyacrylamide hydrogels with different substrate stiffnesses were prepared according to the established sandwich protocol described by Tse and Engler (2010). Glass slides were treated with dichlorodimethylsilane (Merck, cat. no. 8034521000) for 5 min to create a hydrophobic surface that prevents polyacrylamide attachment. Next, coverslips of 15 mm diameter were treated with 3-aminopropyltriethoxysilane (Merck, cat. no. A3648) and glutaraldehyde. The resulting amino-silanated coverslips were air dried. Acrylamide (40%) and bis-acrylamide (2%) were mixed together in various relative concentrations to tune the resulting elasticities. Solutions of acrylamide/bis-acrylamide were crosslinked via a free radical polymerization reaction using ammonium persulfate and tetramethyldiethylenediamine. Immediately after the addition of crosslinkers, 20 µl of acrylamide/bis-acrylamide solution was sandwiched between the glass slide and the amino-silanated coverslip. All polyacrylamide gels were allowed to completely crosslink at room temperature (RT) for 2 h. Polyacrylamide gels were then washed twice in HEPES buffer (50 mM, pH 8.5) and sterilized with ultraviolet (UV) light inside a biological safety cabinet. To allow adhesion, polyacrylamide gels were covalently crosslinked with human fibronectin. A solution of 0.2 µg/µl sulfosuccinimidyl-6-(4 -azido-2-nitrophenylamino)-hexanoate (sulfo-SANPAH; Merck, cat. no. 803332-50MG) was freshly prepared in HEPES buffer with 1% dimethylsulfoxide. The sulfo-SANPAH solution was placed on gels and activated using 1 J of 254 nm UV light (Stratagene UV crosslinker). Afterwards, polyacrylamide gels were washed once with HEPES buffer and incubated with 10 µg/ml fibronectin in HEPES buffer overnight at 4°C. Polyacrylamide gels coated with fibronectin were used within 1 week of preparation to ensure mechanical integrity.

### Transient transfection

Transient transfection was performed using FUGENE HD (Promega) according to the manufacturer's instructions. Plasmids encoding GFP–hamster vimentin 1–138 and NAGTI (MGAT1)–GFP (NAGFP) were provided by Prof. Viki Allan (University of Manchester).

### Generation of cell lines stably expressing GORAB

All cDNA sequences were cloned into pXLG3 MCS [a modified version of pXLG3 with an expanded multiple cloning site (MCS); provided by Prof. Peter Cullen, University of Bristol, UK] for lentivirus production. GORAB was tagged at the amino terminus with BirA*, mApple or GFP by cloning into the SpeI and XhoI or BamHI sites of the pXLG3 MCS. Lentiviruses were prepared as described next, based on a previously described method (Salmon and Trono, 2007). 24 h after seeding, HEK293 LTV cells (2.5×10^6^ cells per 10 cm dish) were co-transfected with 6 µg of the appropriate pXLG3 construct and 4.5 g psPAX2 (Addgene #12260) and 3 µg pM2G (Addgene #12259) vectors (packaging and envelope vectors, respectively) using 27 µl of polyethylenemine mix (Merck, cat. no. 408727). 24 h after transfection, 100 µl of 1 M sodium butyrate was added. After 8 h, the medium was changed to fresh DMEM supplemented with 10% HyClone FBS (cat. no. SH30071) and 1 mM L-glutamine. 72 h after initial transfection, the medium containing lentivirus was gathered, centrifuged at 2700 ***g*** for 10 min at RT and the supernatant was filtered through a 0.45 µm filter (EMD Millipore, Billerica, MA, USA). The lentivirus-containing medium was snap frozen in 1 ml aliquots and stored at −80°C. For lentiviral transduction, cells were plated in 10 cm dishes containing 1×10^6^ cells and incubated with 1–3 ml of filtered medium containing lentivirus for 3 days in antibiotic-free medium. The efficiency of transduction was validated by immunofluorescence microscopy. For stable transduction, cells were trypsinised and seeded into a new 10 cm dish at 1:200,000 dilution and grown in fully supplemented DMEM for a period of 10 to 14 days until distinct cell colonies were formed. The cell medium was removed, cells were washed with DMEM and pre-warmed trypsin, and incubated for 3 min at 37°C. Colonies were picked using a sterile tip and propagated. The expression levels of tagged GORAB proteins were validated by immunofluorescence microscopy and western blotting.

### Generation of GORAB KO MEFs using CRISPR/Cas9

GORAB was targeted using the Custom All-In-One lentivirus system [Applied Biological Materials (abm)]. Lentiviruses containing guide RNAs targeting GORAB were created in HEK293 Lenti-X cells (Takara, 31966021) plated on 10 cm dishes the day before transfection. For each dish, 7 µg of each lentiU6-mGORAB gRNA plasmid alone (abm, 224111140595; gRNA1 target 1–9, 5′-GGATTGGGCGGGCTTCTCTG-3′; gRNA2 target 2–76, 5′-TTCACAGGAATTCGACGCTG-3′; gRNA3 target 3–284, 5′-AGGTAGCTTTCCATCGCCAG-3′), or all three in combination (2 µg of each gRNA plasmid), 3 µg of psPAX2 packing plasmid and 2 µg of pM2G envelope plasmid were transfected into HEK293 Lenti-X cells using 30 µl of Fugene-HD (Promega) in antibiotic-free DMEM. The medium was replaced the day after and the virus-containing medium was collected the following day and filtered through a 0.44 µm syringe filter unit, and 5 ml was used for transduction of WT or vimentin KO MEFs with a 1:1 ratio of medium containing virus to regular medium. The following day, the medium was replaced with fresh medium containing puromycin (2 µg/ml), and cells were selected by culturing in medium containing antibiotic for 7 days. GORAB KO cells were diluted to single cells in 24-well plates. The GORAB KO data shown in the paper were generated with a clone generated using gRNA1. Similar results were obtained using gRNA3. The vimentin-GORAB double KO cells were not able to proliferate from single cells and thus the double KO cells were used as a mixed population of cells obtained from the initial transduction.

### Proximity-dependent biotinylation

The proximity biotinylation method was adapted from Roux et al. (2012). HeLaM or human skin fibroblast cells stably expressing GORAB–BirA were incubated for 24 h in DMEM supplemented with 10% HyClone FBS, 1 mM L-glutamine, penicillin-streptomycin mix and 50 µM biotin. After three PBS washes, 4×10^7^ cells were lysed at 25°C in 1 ml lysis buffer [50 mM Tris-Cl pH 7.4, 0.5 M NaCl, 0.4% SDS, 5 mM EDTA, 1 mM dithiothreitol and protease inhibitor cocktail (Calbiochem)] and sonicated (Bioruptor, Diagenode, Belgium) as per the manufacturer's instructions. Triton X-100 was added to a final concentration of 2%. After further sonication, an equal volume of cooled (4°C) 50 mM Tris-Cl pH 7.4 was added before additional sonication (subsequent steps at 4°C) and centrifugation at 15,000 ***g***. Supernatants were incubated with 500 μl pre-equilibrated streptavidin-Dynabeads (MyOne Steptavadin C1, Thermo Fisher Scientific) overnight. Beads were collected and washed twice for 8 min at 25°C (all subsequent steps at 25°C) in 1 ml wash buffer 1 (2% SDS in dH_2_O). This step was repeated once with wash buffer 2 (50 mM HEPES pH 7.5, 0.1% deoxycholate, 1% Triton X-100, 500 mM NaCl and 1 mM EDTA), once with wash buffer 3 (10 mM Tris-Cl pH 8.1, 250 mM LiCl, 0.5% NP-40, 0.5% deoxycholate and 1 mM EDTA) and twice with wash buffer 4 (50 mM Tris-Cl pH 7.4 and 50 mM NaCl). Samples to be analysed by MS were washed twice in 50 mM NH_4_HCO_3_. On-bead tryptic digests were analysed by one-dimensional liquid chromatography (LC)-MS/MS by Sanford-Burnham Proteomic Facility (La Jolla, CA, USA) using the following procedure. 4 μl of Tris(2-carboxyethyl)phosphine (TCEP) was added to 200 µl of beads/50 mM ammonium bicarbonate suspension mix and proteins were reduced at 40°C for 30 min. Iodoacetamide was added to 20 mM and proteins were alkylated for 30 min at RT in the dark. MS-grade trypsin was added (1:20 ratio) for overnight digestion at 37°C. After digestion, magnetic beads were removed by centrifugation. Formic acid was added to the peptide solution to 2%, followed by desalting and online analysis of peptides by high-resolution, high-accuracy LC-MS/MS, consisting of a Michrom HPLC, a 15-cm Michrom Magic C18 column, a low-flow ADVANCED Michrom MS source and a LTQ-Orbitrap XL (Thermo Fisher Scientific). A 120-min gradient of 10–30% buffer B (0.1% formic acid, 100% acetonitrile) was used to separate the peptides. The total liquid chromatography time was 141 min. The LTQ-Orbitrap XL was set to scan precursors in the Orbitrap followed by data-dependent MS/MS of the top four precursors. Raw LC-MS/MS data were submitted to Sorcerer Enterprise (Sage-N Research, Milpitas, CA, USA) for protein identification against the ipi.HUMAN.vs3.73 protein database, which contains semi-tryptic peptide sequences allowing up to two missed cleavages. A molecular mass of 57 Da was added to all cysteines to account for carboxyamidomethylation. Differential search included 16 Da for methionine oxidation and 226 Da on the N-terminus and lysines for biotinylation. Search results were sorted, filtered, statically analysed and displayed using PeptideProphet and ProteinProphet (Institute for Systems Biology, Seattle, WA, USA) (Nesvizhskii et al., 2003). The minimum trans-proteomic pipeline probability score for proteins was set to 0.95 to assure a trans-proteomic pipeline error rate of lower than 0.01. The relative abundance of each identified protein in different samples were analysed by QTools, an open source in-house-developed tool for automated differential peptide/protein spectral count analysis (Brill et al., 2009).

### Preparation of cell lysates

MEFs were washed three times in PBS and lysed in ice-cold lysis buffer (50 mM Tris-HCl, pH 7.4, 150 mM NaCl, 1% Triton X-100 and 1% protease inhibitor cocktail; 200 µl/well in 6-well plates) and incubated on ice for 30 min. The lysate was cleared by centrifugation at 16,000 ***g*** for 10 min at 4°C and the protein concentration was measured using the Bradford assay.

### Immunoblotting

For immunoblotting, 40–80 µg of cell lysate was loaded onto 10% gels for SDS-PAGE, transferred onto nitrocellulose membranes, followed by blocking in 5% non-fat dried milk in PBS supplemented with 0.15% Tween 20 for 1 h at RT and overnight incubation with primary antibodies at 4°C. HRP-conjugated antibodies were used as secondary antibodies with 1 h incubation at RT. Protein bands were visualized using SuperSignal West Femto substrate (Thermo Fisher Scientific) on a Chemidoc imager (Bio-Rad).

### Immunofluorescence microscopy

Cells were fixed in 3.7% paraformaldehyde (PFA) in PBS for 20 min at 37°C. Then, cells were washed two times with PBS, quenched with 1 M glycine and permeabilized by incubation in 0.25% Triton X-100 in PBS for 5 min at RT. After three washes with PBS, the cells were incubated for 1 h in blocking solution (0.2% saponin, 1% FBS and 0.5% BSA in PBS) followed by primary antibody incubation at RT for 1 h 30 min. Coverslips were then washed three times in PBS for 5 min and incubated with the secondary antibodies supplemented with DAPI (200 ng/ml) for 1 h at RT, washed three times with PBS for 5 min, n dried and mounted on slides using Prolong mounting solution. Images were acquired using an Olympus BX60 upright microscope equipped with a MicroMax cooled, slow-scan CCD camera (Princeton Instruments, Acton, MA, USA) driven by Metaview software (University Imaging Corporation, West Chester, PA, USA). Images were processed using ImageJ/Fiji software (MacBiophotonics, Bethesda, MD, USA). For confocal and stimulated emission depletion (STED) microscopy, images were collected on a Leica TCS SP8 AOBS inverted gSTED microscope with the following confocal setting: pinhole 1 Airy unit, scan speed 400 Hz unidirectional and format 2048×2048; Alexa Fluor 488: 498–542 nm; Alexa Fluor 594: 564–619 nm; Alexa Fluor 647:646–713 nm; using the 490 nm, 555 nm and 635 nm excitation laser lines and 592 nm, 660 nm and 775 nm depletion laser lines, respectively. STED images were deconvolved using Huygens Professional software (Scientific Volume Imaging).

### Live imaging

Cells were seeded on ibidi Petri dishes in complete medium. Next day, cells were transfected with 2 µg of plasmid encoding the Golgi marker NAGT1 (MGAT1) fused to GFP (NAGFP) (Shima et al., 1997) using FUGENE HD transfection. After 24 h, live imaging depicting the effect of BFA (5 µg/ml; Merck, cat no. B7651-5MG) on Golgi disassembly was performed and images were acquired every minute for a period of 20 min using a 3i spinning disc confocal with a 60× objective. Images showing tubules after BFA treatment or BFA washout were acquired using a Zeiss Cell Discoverer 7 Airyscan 2 confocal microscope with a 40× objective. For quantification purposes, 25 cycles were acquired for each of the different positions selected. Images were processed using ImageJ/Fiji Software.

### Drug treatments

Medium containing BFA at a final concentration of 5 µg/ml was added to MEFs grown on coverslips. Cells were incubated for 5, 10, 20, 30 and 60 min in the presence of BFA, prior to fixation in 4% PFA. For BFA washout experiments, MEFs were treated for 90 min with BFA, washed three times with fresh medium and incubated for an additional 15, 30, 45, 60 and 90 min prior to fixation. For depolymerisation of microtubules, cold medium containing nocodazole (Merck, cat. no. M1404-2MG) at a final concentration of 8 µg/ml was added to MEFs and cells were cultured at 37°C for 2 h before fixation. Nocodazole time course experiments were conducted by incubating with 8 µg/ml nocodazole at 37°C for 10, 20, 30, 60 and 120 min. Nocodazole washout was performed by treating cells for 120 min with 8 µg/ml nocodazole, then washing three times in fresh medium and incubating at 37°C for 10, 20, 30, 60 and 120 min.

### PLA

WT or GO cells were cultured on coverslips for 24 h. PLA was carried out using the Duolink *In Situ* Detection Reagent Red kit (Sigma-Aldrich) following the manufacturer's instructions. Briefly, cells were fixed in 4% PFA in PBS for 10 min at RT, followed by a permeabilization step with 0.2% Triton X-100. Coverslips were blocked with blocking solution for 1 h and then incubated with anti-GORAB and anti-vimentin primary antibodies previously diluted in antibody diluent. Coverslips were then washed with 1× Wash Buffer A two times for 5 min at RT and incubated with PLA probes MINUS and PLUS corresponding to the primary antibodies using Duolink *In Situ* PLA Probe Anti-mouse MINUS (Sigma-Aldrich, DUO92004) and Duolink *In Situ* PLA Probe Anti-rabbit PLUS (Sigma-Aldrich, DUO92002) for 1 h. Next, coverslips were washed two times for 5 min in 1× Wash Buffer A and then incubated with a DNA ligase previously diluted in Ligation buffer for 30 min. Coverslips were washed two times for 5 min in 1× Wash Buffer A and incubated with a DNA polymerase previously diluted in Amplification buffer (Sigma-Aldrich, DUO92008) for 90 min. Unless specified otherwise, all incubations were carried at 37°C in a humidified chamber. Finally, coverslips were washed two times for 10 min in 1× Wash buffer B and 1 min in 0.01× Wash buffer B. Coverslips were mounted with Mounting Medium containing DAPI to detect nuclei. Fluorescence was visualized using the Olympus BX60 upright microscope equipped with a MicroMax cooled, slow-scan CCD camera driven by Metaview software.

### TEM analysis of Golgi ultrastructure

MEF cells were grown on 10 cm dishes until they reached confluency. The samples were fixed by adding double-concentrated fixative directly to the same amount of medium in the culture dish, so that the final fixative was 4% formaldehyde with 2.5% glutaraldehyde in 0.1 M HEPES buffer (pH 7.2). Then, samples were post-fixed with reduced osmium (1% osmium tetroxide with 1.5% potassium ferrocyanide) in 0.1 M cacodylate buffer (pH 7.2) for 1 h, then in 1% uranyl acetate in water overnight. The samples were dehydrated in ethanol series infiltrated with TAAB LV resin and polymerized for 24 h at 60°C. 80-nm sections were cut with a Reichert Ultracut ultramicrotome and observed with a FEI Tecnai 12 Biotwin microscope at 100 kV accelerating voltage. Images were taken with a Gatan Orius SC1000 CCD camera.

### Image analysis and statistical tests

Colocalization, Golgi elements and total cell fluorescence were quantified using ImageJ/Fiji software. Golgi element number was determined by counting the structures/cells above the intensity fluorescence threshold that had been selected based on WT MEF cells. Total intensity fluorescence was normalized for cell area. Statistical analyses were conducted using GraphPad Prism software (La Jolla, CA, USA). The D'Agostino–Pearson and Shapiro–Wilk tests were used for comparison of the distribution of data with a Gaussian distribution. Depending on the result, an unpaired two-tailed *t*-test or Mann–Whitney test was performed. In the case of an unpaired two-tailed *t*-test, equality of variances between two groups was tested with an *F*-test. For multiple group comparisons, a one-way or two-way ANOVA followed by Dunnett's test was performed. Statistical significance cut-offs were set as follows: *P≤0.05, **P<0.01, ***P<0.001 and ****P<0.0001.

## Supplementary Material

10.1242/joces.260577_sup1 Supplementary information
