# Supplementary material for: Vimentin intermediate filaments provide structural stability to the mammalian Golgi complex
Source: J Cell Sci. 2023 Oct 18;136(20):jcs260577. doi: 10.1242/jcs.260577 (PMC10617613; doi:10.1242/jcs.260577)
Supplement: Supplementary information [file joces-136-260577-s1.pdf]

FIGURE S1

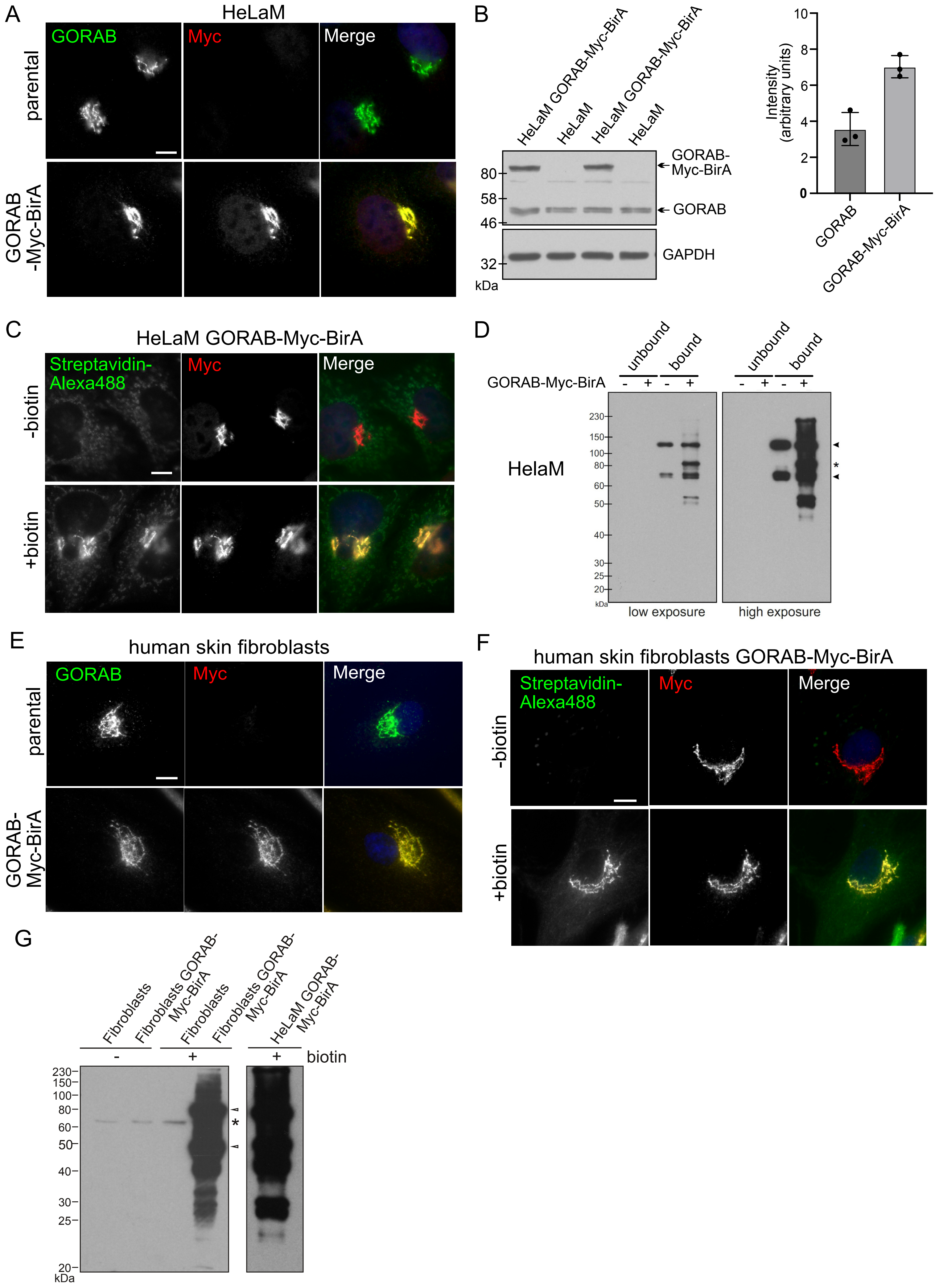

Journal of Cell Science • Supplementary information

Fig. S1. Subcellular localization of GORAB and GORAB-Myc-BirA in HelaM cells and human skin fibroblasts. **A)** Immunofluorescence of the parental HeLaM cell line and the selected HeLaM BIOID-GORAB cell line. Co-localisation analysis using a GORAB antibody recognising endogenous GORAB and GORAB-Myc-BirA and a Myc antibody recognising GORAB-Myc-BirA only. Scale bar, 10 µm; **B)** Left, Western blotting of parental HeLaM cells or HeLaM cells stably expressing GORAB-Myc-BirA with antibodies to GORAB or GAPDH. Two lanes are shown for each cell type. Right, quantitation of GORAB signal for endogenous protein (GORAB) or the over-expressed tagged form (GORAB-Myc-BirA), expressed as intensity in arbitrary units. **C)** Co-localisation analysis in HeLaM cells of streptavidin-Alexa488, recognising biotinylated proteins, and either GORAB or Myc antibody. Cells were grown in standard medium or medium supplemented with 50 µM biotin for 24 h prior to fixation. Scale bar, 10 µm; **D)** Western blot analysis of biotinylated proteins in HeLaM and HeLaM GORAB-Myc-BirA cell lines. HeLaM and HeLaM GORAB-Myc-BirA cell lines were grown in medium supplemented with 50 µM biotin for 24 h prior to cell lysis. Biotinylated proteins were pulled down using streptavidin-coupled beads. Unbound (50%) and bound (50%) fractions were subjected to SDS-PAGE and biotinylated proteins were detected by streptavidin-HRP. A specific band corresponding to GORAB-Myc-BirA is marked with an asterisk while endogenously biotinylated proteins are marked with an arrow; **E)** Immunofluorescence of wild-type human skin fibroblasts and fibroblasts transduced with a lentivirus expressing GORAB-Myc-BirA. Co-localisation analysis using a GORAB antibody recognising endogenous GORAB and GORAB-Myc-BirA and a Myc antibody recognising GORAB-Myc-BirA only. Scale bar, 10 µm; **F)** Immunofluorescence of wild-type fibroblasts and fibroblasts transduced with a lentivirus expressing GORAB-Myc-BirA. Co-localisation analysis of streptavidin-Alexa488, recognising biotinylated proteins, and either GORAB or Myc antibody. Cells were grown in standard medium or medium supplemented with 50 µM biotin for 24 h prior to fixation. Scale bar, 10 µm; **G)** Western blot analysis of biotinylated proteins in wild-type fibroblasts or fibroblasts stably expressing GORAB-Myc-BirA. Cells were grown in standard medium or medium supplemented with 50 µM biotin for 24 h prior to cell lysis. Cell lysates were subjected to the SDS-PAGE and biotinylated proteins were detected by streptavidin-HRP. The band corresponding to GORAB-Myc-BirA is marked with an asterisk while endogenously biotinylated proteins are marked with an arrow. Right panel: analysis of biotinylated proteins pulled-down from the HeLaM GORAB-Myc-BirA cell line, taken from panel C.

Journal of Cell Science • Supplementary information

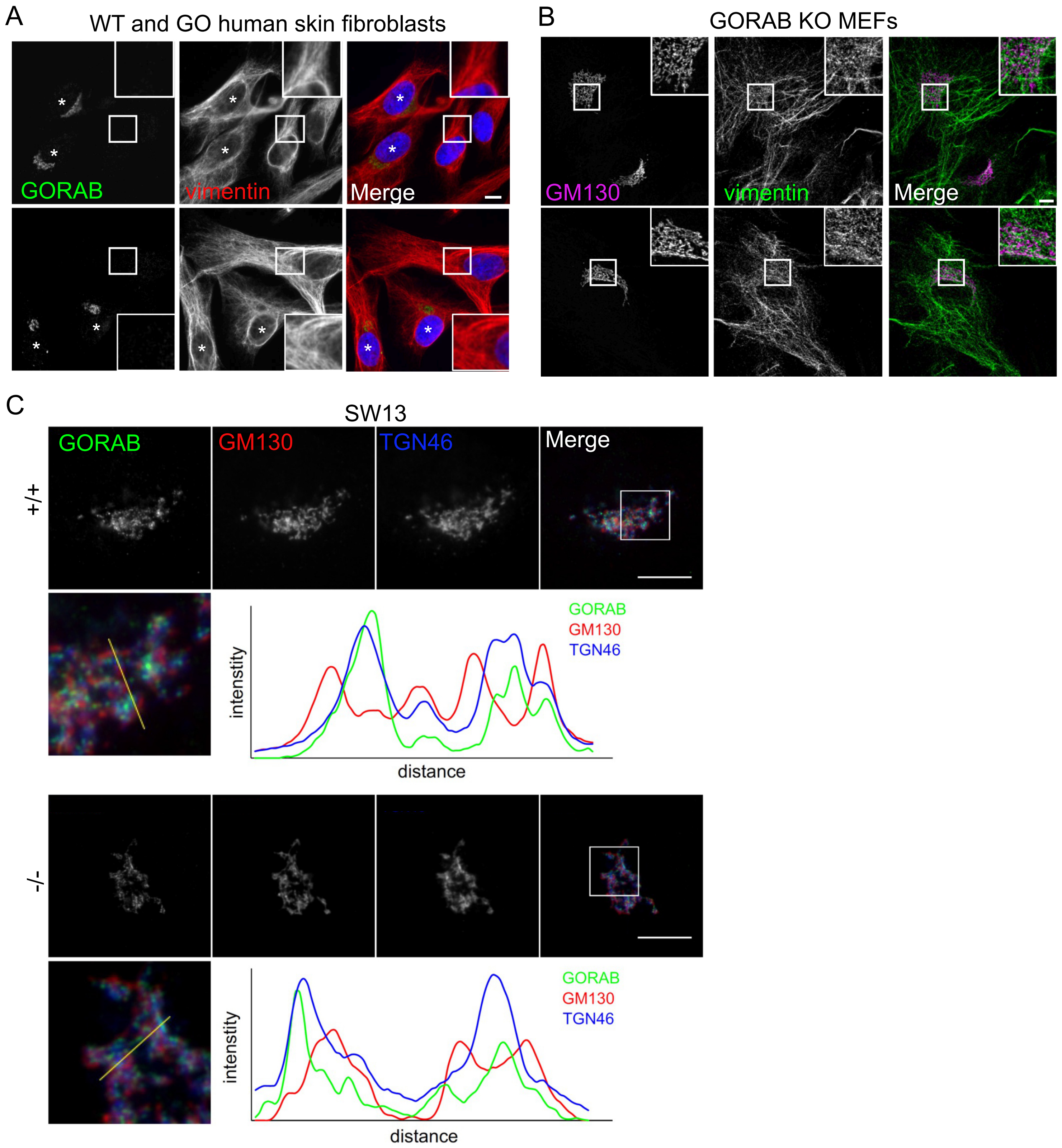

Fig. S2. **Structure of vimentin filaments is indistinguishable between WT and GORAB-deficient fibroblasts. A)** Immunofluorescence of co-culture of human skin fibroblasts derived from a healthy individual or GO patient containing a L206P GORAB variant that is not targeted to the Golgi apparatus. Scale bar, 10 µm; **B)** Immunofluorescence of GM130 colocalisation with vimentin filaments in GORAB KO MEFs. Scale bar, 10 µm; **C)** Immunofluorescence of co-localisation between GORAB, GM130 and TGN46 in SW13$^{+/+}$ and SW13$^{-/-}$ cells. Yellow lines depict the areas taken for calculating RGB fluorescence intensity profile plots. Scale bar, 10 µm.

Journal of Cell Science • Supplementary information

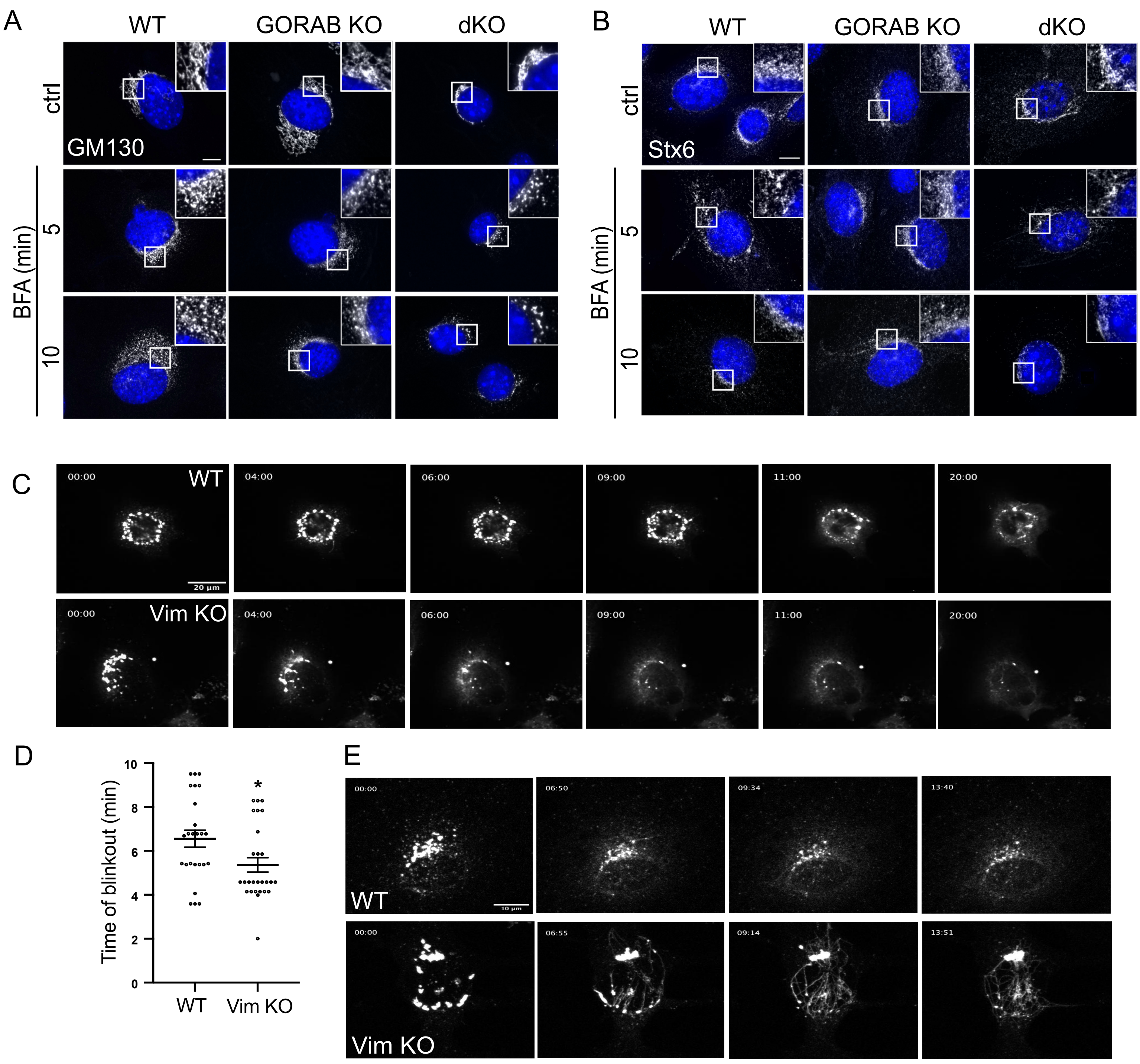

Fig. S3. Golgi disassembly in KO MEFs treated with BFA. A,B) Immunofluorescence of GM130 (A) and Stx6 (B) after treatment of WT, GORAB KO and dKO MEFs for 5 or 10 min with BFA. Scale bar, 10 µm. **C)** Golgi disassembly in NAGFP-expressing WT and vimentin KO MEFs was induced by treatment with 5 µg/ml BFA. Imaging was performed with a 3i spinning disc microscope. Representative images are shown for the indicated timepoints following BFA addition. Microscope. Scale bar, 20 µm. **D)** Quantification of Golgi disassembly. Blinkout is defined as the first reduction in Golgi fluorescence signal. Comparisons between groups were made with an unpaired t-test. Error bars show SEM. n=25 for WT and n=27 for KO. **E)** Golgi disassembly in NAGFP-expressing WT and vimentin KO MEFs was induced by treatment with 5 µg/ml BFA. Imaging was performed with a Zeiss Airyscan 2 microscope. Representative images are shown for the indicated timepoints following BFA addition. Scale bar, 10 µm.

Journal of Cell Science • Supplementary information

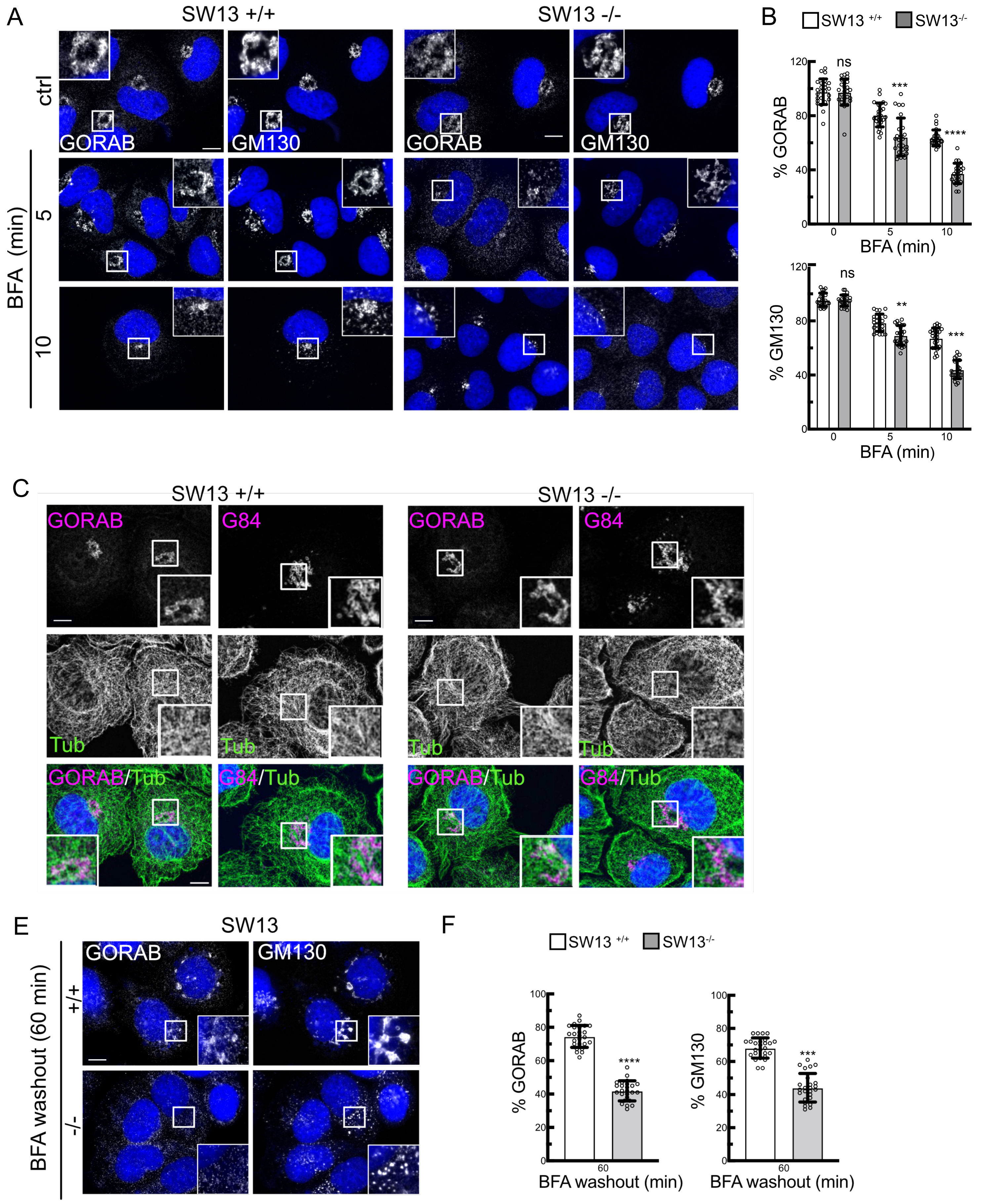

Journal of Cell Science • Supplementary information

Fig. S4. Golgi fragmentation and reassembly in SW13$^{+/+}$ and SW13$^{-/-}$ cells treated with BFA. A) Immunofluorescence analysis of Golgi disassembly indicated using antibodies to GORAB and GM130 at 5 and 10 min BFA treatment in SW13+/+ and SW13-/- cells. Scale bar, 10 µm**; B)** Analysis of fluorescence intensity of GORAB and GM130 at 5 and 10 min after BFA treatment compared to untreated cells. Comparisons between groups were made with an unpaired t-test. Error bars show SEM. n=30; **C)** Immunofluorescence of GORAB and GM130 60 min after BFA washout. Scale bar, 10 µm; **D)** Analysis of fluorescence intensity of GORAB and GM130 60 min after BFA washout. **E)** Colocalisation analysis of GORAB and Golgin-84 with microtubules at steady state. **F)** Quantification of GORAB and GM130 reassembly in SW13+/+ and SW13-/- cells. Comparisons between groups were made with an unpaired t-test. Error bars show SEM. n=30. Scale bar, 10 µm.

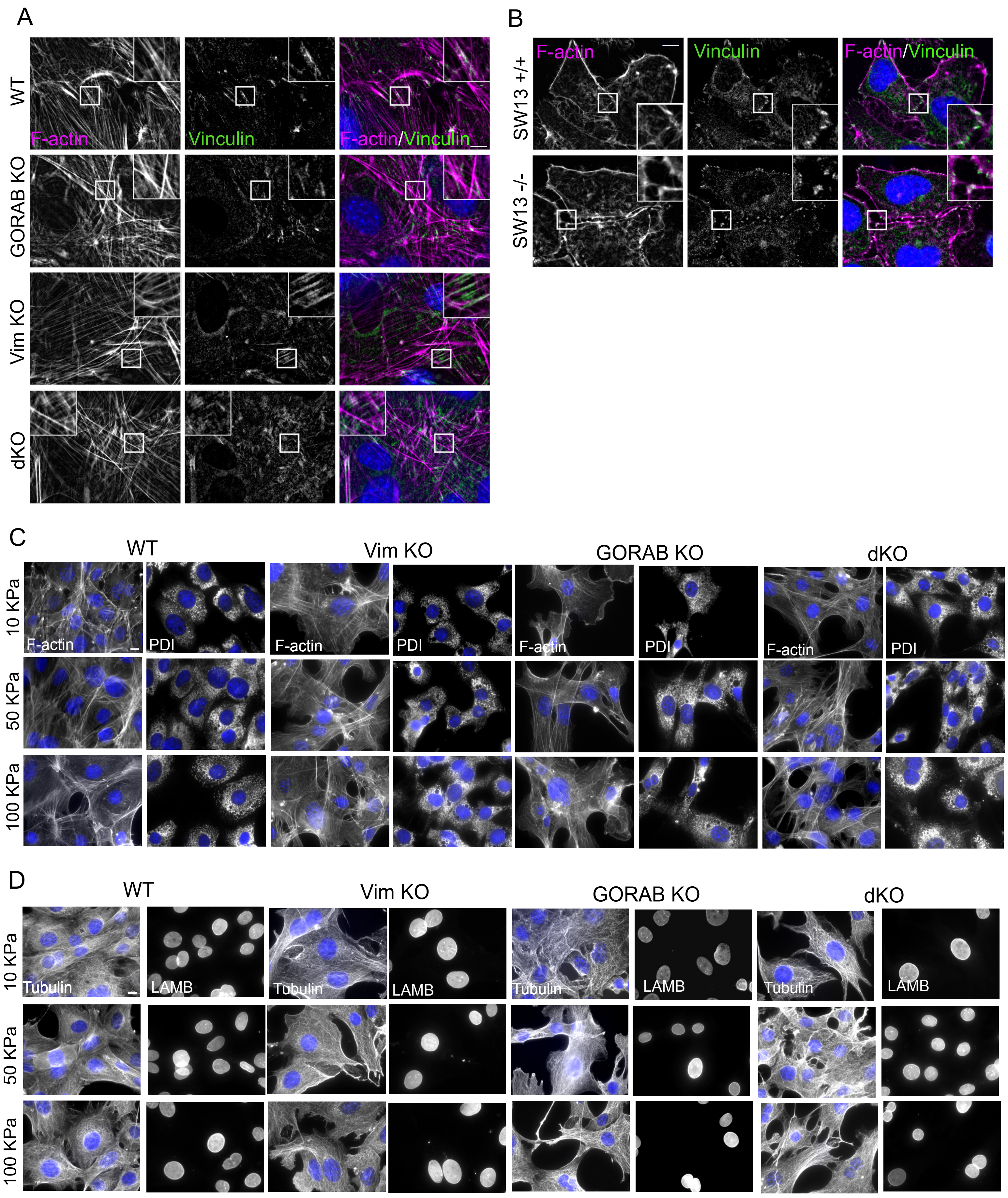

Fig. S5. Cytoskeleton and organelle organisation in the KO MEFs. A) Immunofluorescence of F-actin and focal adhesions (FA, vinculin) in GORAB KO, Vim KO and dKO MEFs compared to WT MEFs. Scale bar, 10 µm; **B)** Immunofluorescence of F-actin and focal adhesions (FA, vinculin) in $SW13^{+/+}$ and $SW13^{-/-}$ cells. Scale bar, 10 µm. **C,D)** Cells were plated on elastic matrigel coated coverslips of the indicated stiffness for 48 h prior to fixation and labelling for F-actin and PDI (C) or tubulin and lamin B (D). Scale bar, 10 µm.

Journal of Cell Science • Supplementary information

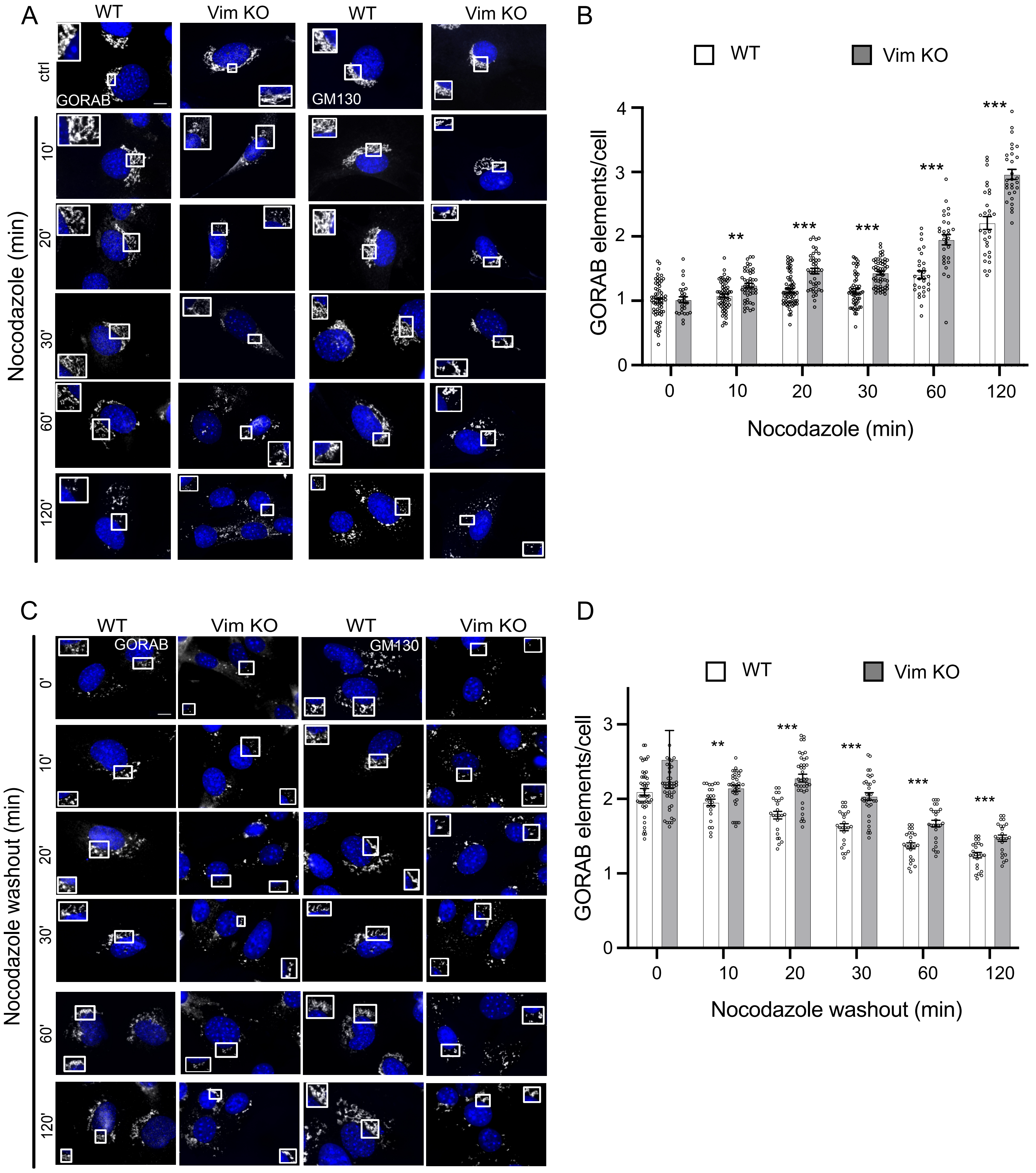

Fig. S6. Nocodazole disassembly and reassembly in vimentin KO MEFs. A) WT and vimentin KO MEFs were incubated with nocodazole (8 µg/ml) for the indicated times at 37˚C, prior to fixation and immunostaining with antibodies to GORAB and GM130. Scale bar, 10 µm; **B)** Quantification of GORAB disassembly in WT and Vim KO MEFs. Comparison between groups was performed with an unpaired t-test. Error bars show SEM. n=30; **C)** WT and vimentin KO MEFs were incubated with nocodazole (8 µg/ml) for 2 h at 37˚C, washed, and incubated in medium lacking nocodazole for the indicated times before fixation and labelling for GORAB and GM130. Scale bar, 10 µm; **D)** Quantification of GORAB reassembly in WT and Vim KO MEFs. Comparison between groups was performed with an unpaired t-test. Error bars show SEM. n=30.

Journal of Cell Science • Supplementary information

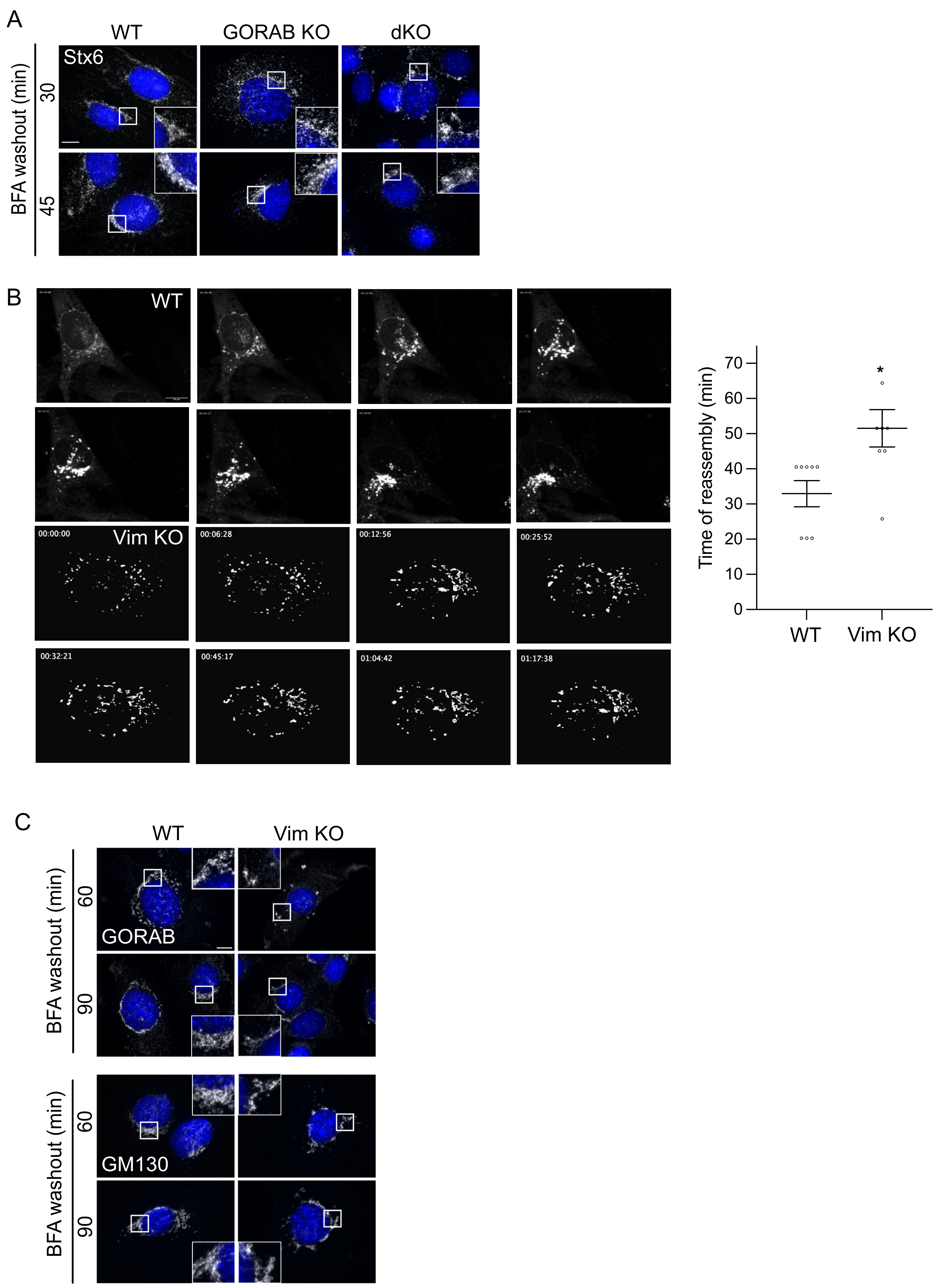

Fig. S7. Golgi disassembly upon BFA washout in the KO MEFs. A) Immunofluorescence analysis of Stx6 reassembly 30 and 45 min after BFA washout in WT, GORAB KO and dKO MEFs. Scale bar, 10 µm; **B)** Golgi disassembly in NAGFP-expressing WT and vimentin KO MEFs was induced by treatment with 5 µg/ml BFA for 90 min, following by washing into fresh medium. Left, Golgi reassembly was imaged live, starting at 9 min post-washout. Imaging was performed with a 3i spinning disc microscope. Representative images are shown for the indicated timepoints post-washout. Scale bar, 10 µm. Right, quantification of Golgi reassembly. Reassembly was defined as the point where fluorescence in the Golgi area increased 2-fold over the starting signal. Comparison between groups was performed with an unpaired t-test, n=8. **C)** Immunofluorescence analysis of GORAB reassembly 60 and 90 min after BFA washout in WT and Vim KO MEFs. Scale bar, 10 µm.

Table S1. List of unique proteins identified by a proximity-dependent biotinylation assay with HeLaM BIOID-GORAB cells. Proteins also identified in the control BIOID pull-down performed by Roux *et al.** are marked in red. Proteins identified both by BIOID-GORAB in this experiment and by BIOID-Lamin A performed by Roux *et al.* are marked in blue. Unique proteins identified in parental HeLaM cells and proteins identified both in HeLaM and HeLaM BIOID-GORAB cells are not shown.

Gene symbol	*Protein name*	*Spectral counts**
GORAB	RAB6-interacting golgin	402
FLNA	Filamin-A	123
AHNAK	Neuroblast differentiation-associated protein	119
VIM	Vimentin	104
HIST1H1E	Histone H1.4	39
HIST1H1D	Histone H1.3	38
KRT4	Keratin, type II cytoskeletal 4	27
KRT6	Keratin, type II cytoskeletal 6	22
KRT18	Keratin, type I cytoskeletal 18	20
CTTN	Src substrate cortactin	12
SNORD38B	RPS8;SNORD55 40S ribosomal protein S8	11
GNL3	Guanine nucleotide-binding protein-like 3	10
COPG2	Coatomer subunit gamma-2	9
PRSS3	Protease serine 2, trypsin-3	7
CRK	Adapter molecule crk	6
	mRNA clone with similarity to L-glycerol-3-phosphate:(NAD) oxidoreductase and albumin gene sequences	6
VAMP3	Vesicle-associated membrane protein 3	6
C1orf35	Multiple myeloma tumor-associated protein 2	5
CDK16	Cyclin-dependent kinase 16	5
RPL23A	60S ribosomal protein L23a	3
FASN	Fatty acid synthase	3
CPD	Carboxypeptidase D	3
SCYL1	N-terminal kinase-like protein 1	3
SLC38A2	Sodium-coupled neutral amino acid transporter 2	3
TAGLN2	Transgelin-2	3
HIST2H2BE	Histone H2B type 2-E	2
PCBP2	poly(rC)-binding protein 2	2
PDAP1	28 kDa heat- and acid-stable phosphoprotein	2
EIF5	Eukaryotic translation initiation factor 5	2
DSG1	Desmoglein-1	2
WWOX	WW domain-containing oxidoreductase	2
ATG4A	Cysteine protease ATG4A	2
SCYL3	Protein-associating with the carboxyl-terminal domain of ezrin	2
CLINT1	Clathrin interactor 1	2
CCT8	T-complex protein 1 subunit theta	2
MKL2	MKL/myocardin-like protein 2	2
RPL11	60S ribosomal protein L11	2
	cDNA FLJ60082, weakly similar to Uro-adherence factor A (Fragment)	2
FOXN1	Forkhead box protein N1	1
DSP	Desmoplakin	1
RPS24	40S ribosomal protein S24	1
SULT1C4	Sulfotransferase 1C4	1
	Ig heavy chain V-III region TRO	1

*Total number of spectral counts per protein

Journal of Cell Science • Supplementary information

Table S2. List of unique protein identified by proximity-dependent biotinylation with human fibroblasts expressing BIOID-GORAB.

Proteins also identified in the control BIOID pull-down performed by Roux *et al.** are marked in red. Proteins identified both by BIOID-GORAB in this experiment and by BIOID-Lamin A performed by Roux *et al.* are marked in blue. Proteins identified both in HeLaM BIOID-GORAB cells and fibroblasts BIOID-GORAB cells are underlined. Unique proteins identified in parental fibroblasts cells and proteins identified both in fibroblasts and fibroblasts BIOID-GORAB cells are not shown.

Gene symbol	***Protein name***	***Spectral counts****
FLNA	Filamin A	305
VIM	Vimentin	279
GORAB	RAB6-interacting golgin	265
AHNAK	Neuroblast differentiation-associated protein AHNAK	139
ACE	Angiotensin-converting enzyme	18
CRK	Adapter molecule crk	15
CTTN	Src substrate cortactin	14
AHNAK2	Protein AHNAK2	8
ANXA2	Annexin A2	7
COPG2	Coatomer subunit gamma-2	6
GOLGA4	Golgin subfamily A member 4; p230	6
ACTB	Actin, cytoplasmic 1	3
RPL29	60S ribosomal protein L29	3
TUBA1C	Tubulin alpha-1C chain	3
WWC3	Protein WWC3	3
MAP1B	Microtubule-associated protein 1B	2
PEA15	Astrocytic phosphoprotein PEA-15	2
EIF5	Eukaryotic translation initiation factor 5	2
CORO1B	Coronin-1B	1
RTN4	Reticulon-4	1
EEF1A1	Elongation factor 1-alpha	1
UBB; RPS27A	UBC ubiquitin and ribosomal protein S27a precursor	1

*Total number of spectral counts per protein

*** Roux, K. J., Kim, D. I., Raida, M. and Burke, B.** (2012). A promiscuous biotin ligase fusion protein identifies proximal and interacting proteins in mammalian cells. *J Cell Biol* **196**, 801-10.

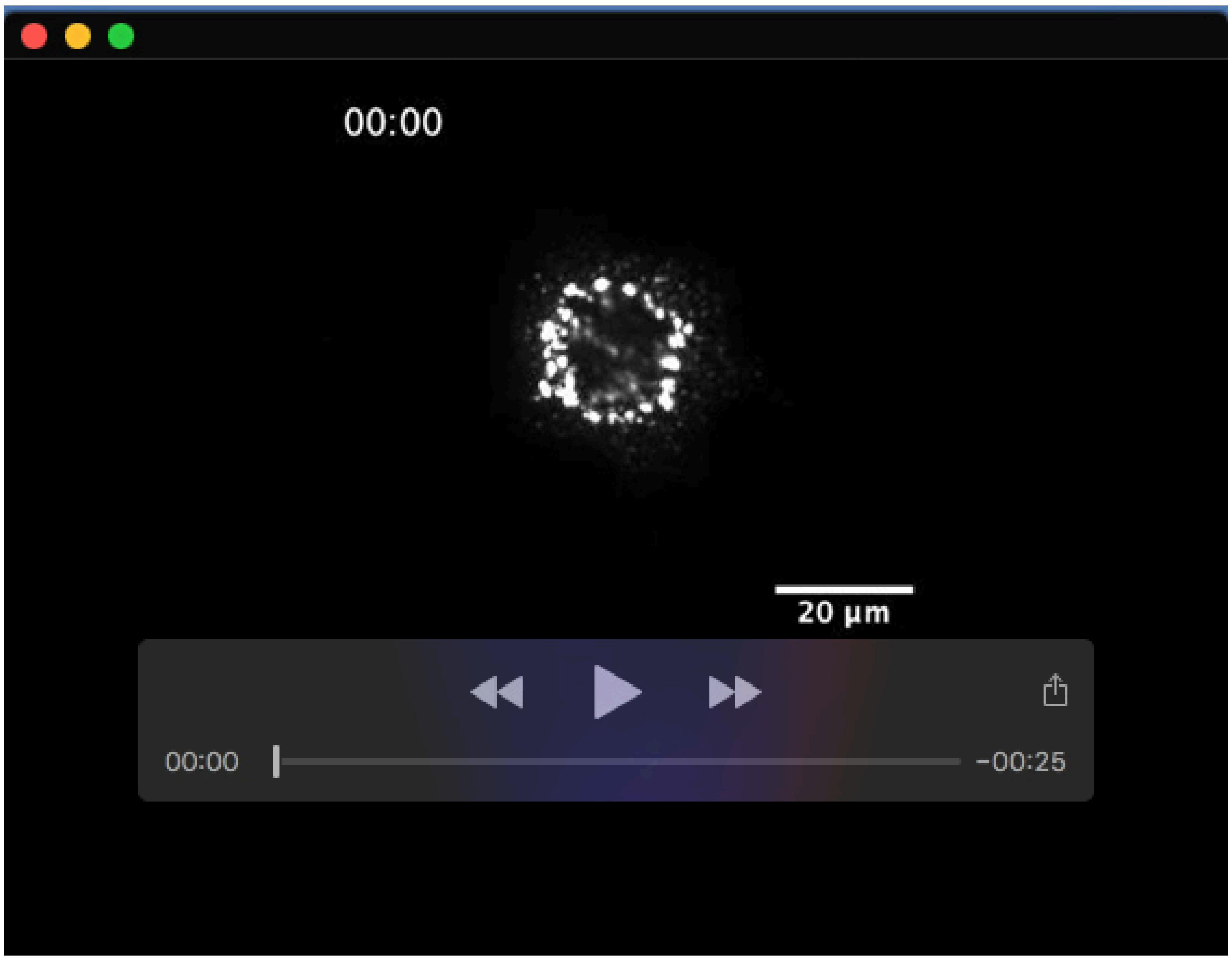

Movie 1. Live imaging of Golgi disassembly in WT MEFs. Related to Fig S3C. WT MEFs expressing NAGFP were treated with 5 µg/ml BFA and imaged using a 3i spinning disc confocal microscope. Movie started immediately after BFA addition. Scale bar, 20 µm.

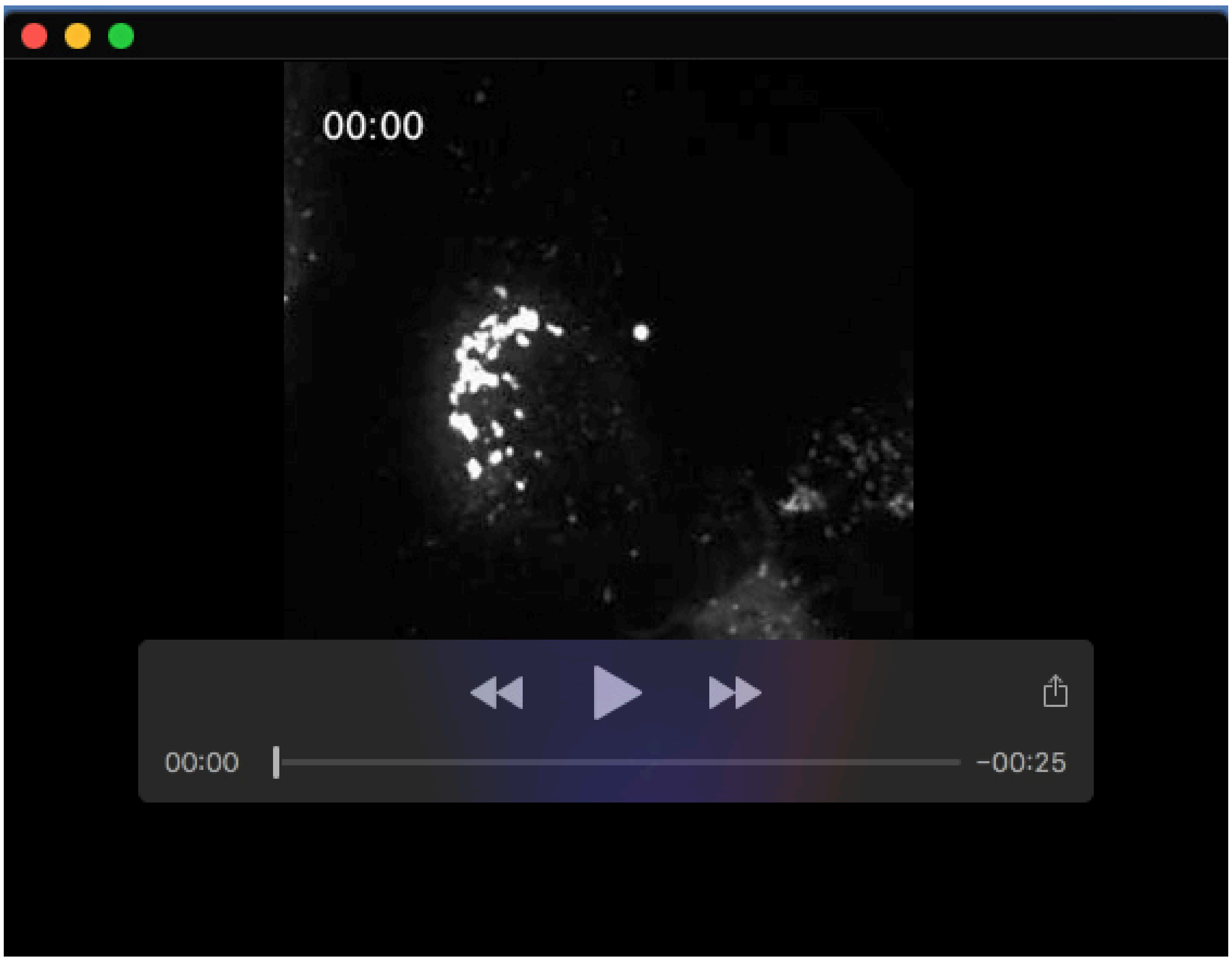

Movie 2. Live imaging of Golgi disassembly in vimentin KO MEFs. Related to Fig S3C. Vim KO MEFs expressing NAGFP were treated with 5 µg/ml BFA and imaged using a 3i spinning disc confocal microscope. Movie started immediately after BFA addition. Scale bar, 20 µm

Journal of Cell Science • Supplementary information

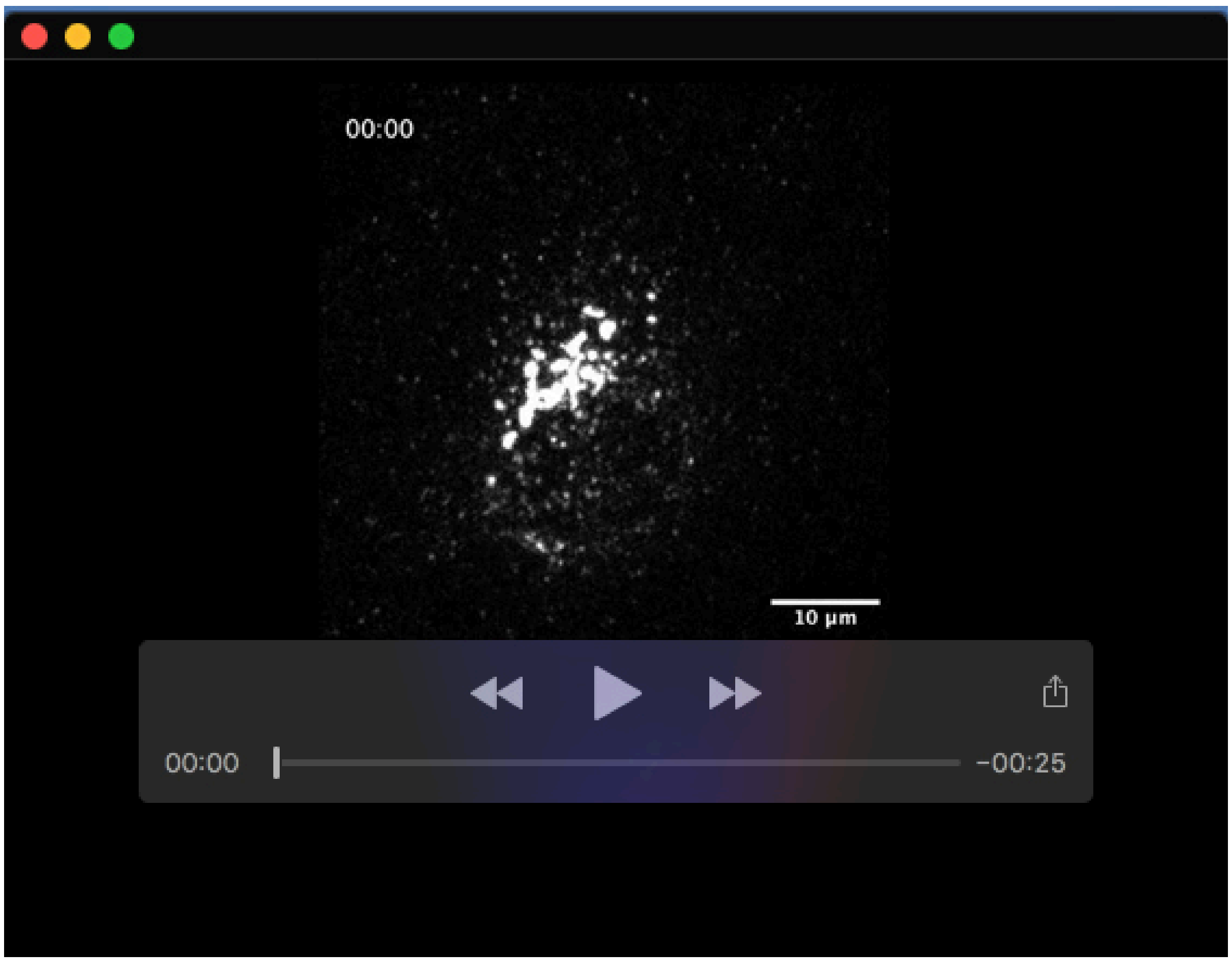

Movie 3. Golgi membrane dynamics upon BFA-induced disassembly in WT MEFs. Related to Fig S3E. WT MEFs expressing NAGFP were treated with 5 µg/ml BFA and imaged using a Zeiss Cell Discoverer 7 Airyscan 2 confocal microscope. Movie started immediately after BFA addition. Scale bar, 10 µm**.**

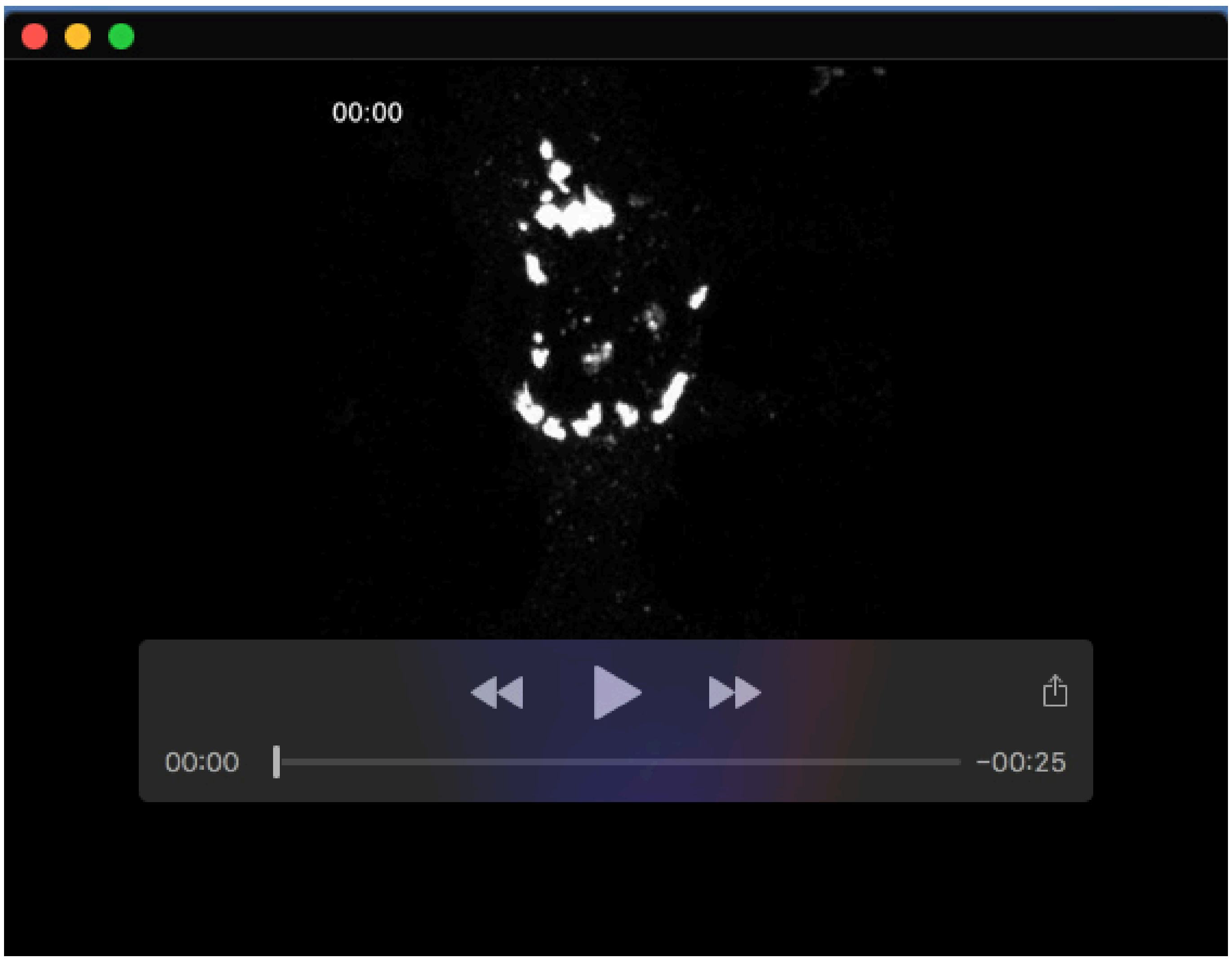

Movie 4. Golgi membrane dynamics upon BFA-induced disassembly in vimentin KO MEFs. . Related to Fig S3E. Vim KO MEFs expressing NAGFP were treated with 5 µg/ml BFA and imaged using a Zeiss Cell Discoverer 7 Airyscan 2 confocal microscope. Movie started immediately after BFA addition. Scale bar, 10 µm.

Journal of Cell Science • Supplementary information

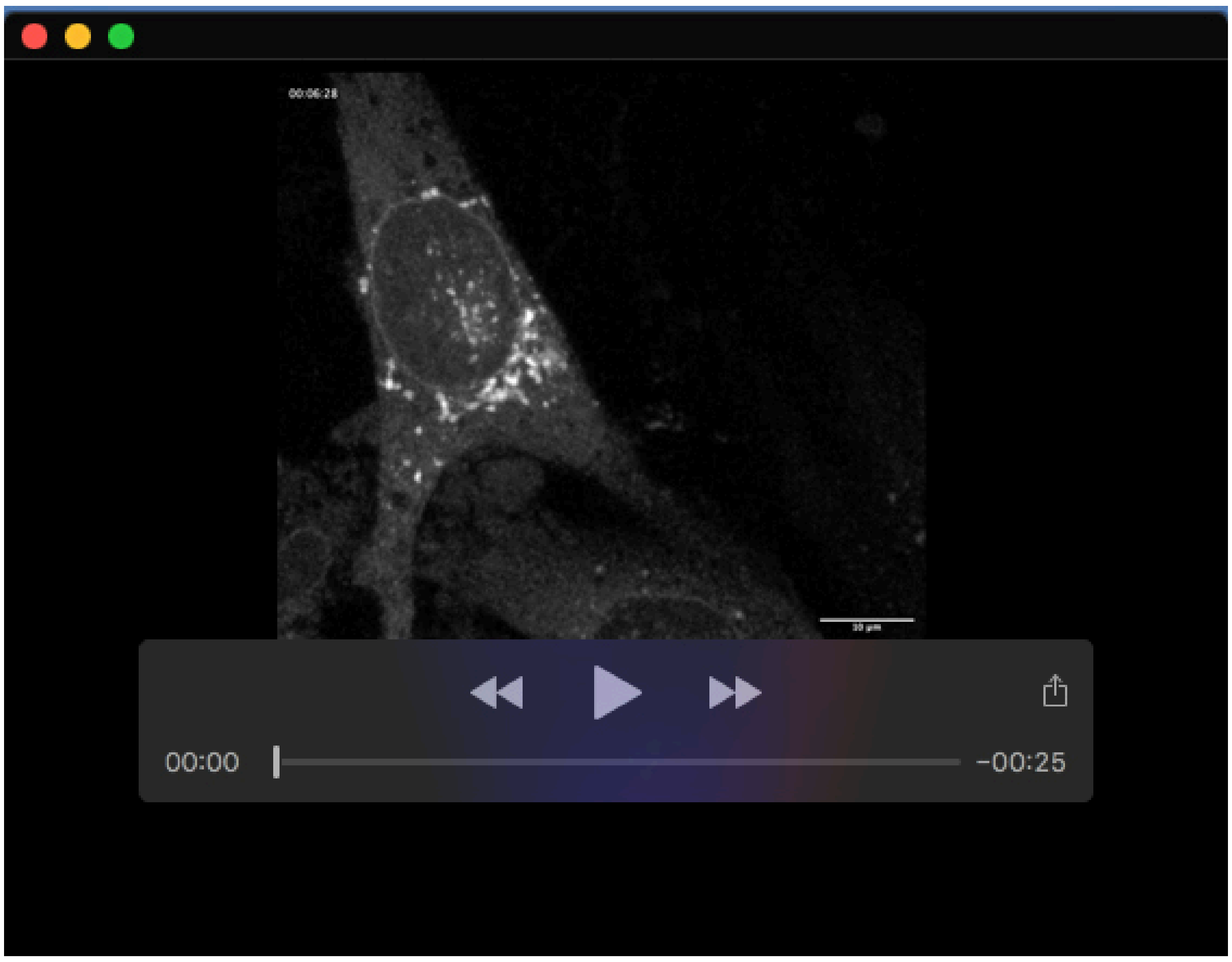

Movie 5. Live imaging of Golgi reassembly in WT MEFs. Related to Fig S7B. Prior to the start of the movie, complete Golgi disassembly was induced in WT MEFs by treatment with 5 µg/ml BFA for 90 min, following by washing into fresh medium. Imaging started at X min post-washout and was captured on a 3i spinning disc confocal microscope. Scale bar, 10 µm.

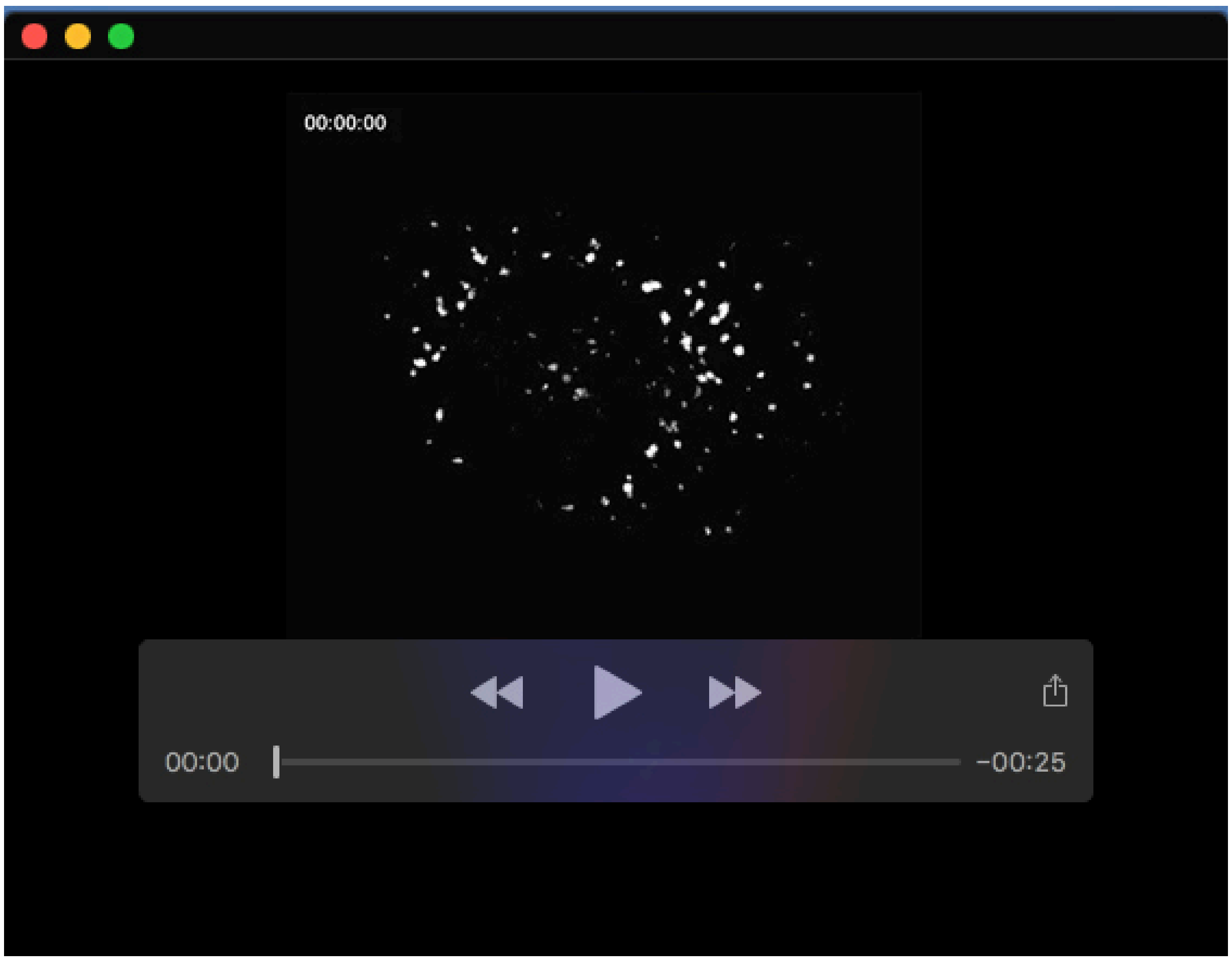

Movie 6. Live imaging of Golgi reassembly in vimentin KO MEFs. Related to Fig S7B. Prior to the start of the movie, complete Golgi disassembly was induced in vimentin KO MEFs by treatment with 5 µg/ml BFA for 90 min, following by washing into fresh medium. Imaging started at X min post-washout and was captured on a 3i spinning disc confocal microscope. Scale bar, 10 µm.

Journal of Cell Science • Supplementary information
