# Supplementary material for: Vimentin intermediate filaments provide structural stability to the mammalian Golgi complex
Source: J Cell Sci. 2023 Oct 18;136(20):jcs260577. doi: 10.1242/jcs.260577 (PMC10617613; doi:10.1242/jcs.260577)
Supplement: Supplementary file 1 [file joces-136-260577_review_history.pdf]

Vimentin intermediate filaments provide structural stability to the mammalian Golgi complex

Teresa Vitali, Rosa Sanchez-Alvarez, Tomasz M. Witkos, Ioannis Bantounas, Marie F. A. Cutiongco, Michal Dudek, Guanhua Yan, Alexander A. Mironov, Joe Swift and Martin Lowe
DOI: 10.1242/jcs.260577

Editor: Giampietro Schiavo

Review timeline
Original submission: 25 August 2022
Editorial decision: 10 October 2022
First revision received: 11 September 2023
Accepted: 18 September 2023

Original submission

First decision letter

MS ID#: JOCES/2022/260577

MS TITLE: Vimentin intermediate filaments provide structural stability to the mammalian Golgi apparatus

AUTHORS: Teresa Vitali, Tomasz M Witkos, Marie F Cutiongco, Guanhua Yan, Alexander A Mironov, Joe Swift, and Martin Lowe
ARTICLE TYPE: Research Article

We have now reached a decision on the above manuscript.

To see the reviewers' reports and a copy of this decision letter, please go to: https://submit-jcs.biologists.org and click on the 'Manuscripts with Decisions' queue in the Author Area. (Corresponding author only has access to reviews.)

As you will see, the reviewers raise a number of criticisms that prevent me from accepting the paper at this stage. They suggest, however, that a revised version might prove acceptable, if you can address their concerns. If you think that you can deal satisfactorily with the criticisms on revision, I would be pleased to see a revised manuscript. We would then return it to the reviewers.

Please ensure that you clearly highlight all changes made in the revised manuscript. Please avoid using 'Tracked changes' in Word files as these are lost in PDF conversion.

I should be grateful if you would also provide a point-by-point response detailing how you have dealt with the points raised by the reviewers in the 'Response to Reviewers' box. Please attend to all of the reviewers' comments. If you do not agree with any of their criticisms or suggestions please explain clearly why this is so.

© 2023. Published by The Company of Biologists under the terms of the Creative Commons Attribution License (https://creativecommons.org/licenses/by/4.0/).

Reviewer 1

Advance summary and potential significance to field

The manuscript by Vitali et al investigates the role of the intermediate filament vimentin on the integrity of the mammalian Golgi ribbon structure. Their studies provide evidence that vimentin filaments are in close proximity to the Golgi resident protein GORAB. Loss of vimentin does not seemingly alter the Golgi ribbon under steady state conditions but affects the Golgi morphology in cells plated on matrices with increased stiffness. Vimentin depletion further altered the dynamics of disassembly and reassembly of the Golgi after brefeldin A treatment. Based on the results, the authors conclude that vimentin stabilizes the Golgi structure.

Whether intermediate filaments mechanically support intracellular organelles such as the Golgi has been an unresolved question. This study directly shows that vimentin provides stability to the integrity of the Golgi ribbon morphology and as such makes an important contribution to the field. The data are clear and of very high quality. The manuscript is well written and should be published in JCS.

Comments for the author

There are only few minor suggestions the authors could consider:

1. The authors show that the MT network appears to be normal under steady-state conditions in vimentin ko cells, but it is not clear if MT dynamics are changed. The Golgi structure and integrity depends on the MT network and MTs physically interact with vimentin filaments to stabilize dynamic MT polymers (PMID: 34145230). As such it is possible that a changed MT turnover affects Golgi stability. The authors could discuss/test the possibility that Golgi ribbon disassembly and reformation dynamics are changed when MTs are depolymerized and reform. Similarly, the authors could stain for MTs in Fig.6. The MT network in vimentin ko cells might be altered depending on the stiffness of the culture matrix, which in turn could affect Golgi integrity.

2. In Fig.3 the authors use EM to evaluate the ultrastructure of Golgi stacks. The number of cisternae per stack is significantly reduced and the length of the cisternae are shorter. Although this is a quite a big change, the author conclude that “GORAB and vimentin do not play a major role in Golgi organization, polarity or positioning under steady state conditions.” Please explain the reasons for this conclusion. By looking at the EM images it also appears to me that the number of vesicles in the Golgi area is substantially increased. Loss of cisternae, shortened cisternae and more vesicles (and the changes in dynamics induced by brefeldin A) may also point to a membrane transport defect. The authors should discuss this possibility.

3. Vimentin knock out mice are normal and do not show an obvious phenotype (PMID: 7954832). Does this mean that the changes in Golgi structure in vimentin depleted cells are irrelevant to the animals?

4. Overexpressed GORAB forms filament-like structures which are dependent on vimentin. The authors conclude that these GORAB filaments assemble on preexisting vimentin IF, which they argue is consistent with a “physical interaction” (page 5 and abstract). The fact that overexpressed GORASP forms filaments that colocalize with vimentin does not necessarily mean that the two proteins physically interact. Similarly, on page 10 it is discussed that GORAB oligomerization is required for vimentin binding. The experiments presented in this manuscript do provide evidence for binding. Fig.S2 merely shows colocalization of overexpressed GORAB with vimentin filaments, but not binding. I suggest changing the text from “binding” to “colocalization”.

Reviewer 2

Advance summary and potential significance to field

In this manuscript, Vitali, Lowe and coll. question the role of vimentin and intermediate filaments in Golgi organization in particular through the function of the GORAB protein.

© 2023. Published by The Company of Biologists under the terms of the Creative Commons Attribution License (https://creativecommons.org/licenses/by/4.0/).

Altogether, this is an interesting study that explores an overlooked role played by microfilaments on the control of Golgi dynamics and localization. Results are in general convincing and well analyzed, and the reported observations would open novel questions in membrane cell biology. In conclusion, I think that this study may be published by the Journal of Cell Science after considering the points raised bellow.

Comments for the author

1. The main question raised by the approach is whether over-expression of GORAB, that modifies strongly its localization, is meaningful. I think it would help a lot to add a few complementation experiments like a rescue of GORAB KO effects (on Golgi organization ?) with a K190 deletion mutant or an immunoprecipitation done at endogenous expression levels.

2. The second main difficulty I have with this study is the impact of substrate stiffness on Golgi organization. The authors are aware of this and discuss it, but I am still puzzled. On the one hand, no effect is seen on a hyper-stiff glass substrate while it is clearly observed when cultivating cells on 100 kPa substrate. For dKO cells, effect is actually seen on all rigidity (to different levels) but not on glass. The authors may like to stain microtubules/actin of cells seeded on substrates with different stiffness and glass to try to understand better what is happening.

To me, this is not central to the study and because the authors have no experiments to explain this different behavior, I am wondering whether the authors should not choose to remove these data and invest on a larger set of experiments to understand this in a later study.

3. The main effect reported upon Vimentin deletion is on Golgi disassembly/reassembly. While it may intuitively be simple to imagine why a loss of a Vimentin scaffold would allow for faster disassembly, it is harder to imagine why reassembly would be slowed down in these conditions. I think it would be important to complement these data with experiment (1) monitoring in real-time Golgi disassembly and (2) tracking the behavior of Golgi proteins that are, in contrast to GM130, quantitatively transported to the ER upon BFA treatment (like a GFP-tagged Golgi enzyme [or simply its trans-membrane domain as often done]).
The presence of tubules, or not, at early stages after BFA addition would be interesting to analyze as well.

4. Still in the frame of the “scaffolding” model proposed by the authors, addition a set of nocodazole/nocodazole washout experiment would be interesting to add as well. Intriguingly, data shown in Figure 3 suggest that Vimentin KO impacts localization of GORAB on mini stacks and the extend of dispersion in the cytosol but this would need quantification, and, as suggested above, real-time experiments.

5. The authors indicate that GORAB can physically associate with IF but to me no such data is presented. As already proposed above, direct interaction may be looked for by the authors, in particular between endogenous (and not overexpressed) GORAB and Vimentin.

6. As a more minor remark, it is quite strange to enter so deeply in the study with many supplementary figures before referring to main figures. I would propose to move Fig S1 as a main figure as it is an important starting point.

Also, in this Figure the authors need to homogenize bioID/BirA nomenclature.

7. Fig 2A : there seem to be a lower expression of GORAB in Vimentin KO cells. Is this significant ?

8. Quantification of Golgi elements was done by counting the number of elements above the intensity threshold of the Golgi in MEFwt cells, is total intensity of the Golgi marker the same in the different conditions? (Figure 2 suggests a lower expression that may impact image analysis)

9. There is a “funny” typo in legend of figure S2 “residual lowe level expression”
Also, “olgomerization” is indicated in the second page of the discussion

© 2023. Published by The Company of Biologists under the terms of the Creative Commons Attribution License (https://creativecommons.org/licenses/by/4.0/).

Reviewer 3

Advance summary and potential significance to field

In the manuscript by Vitali et al, the authors described a set of interesting and, for the most part, well-controlled experiments in support of their hypothesis that vimentin intermediate filaments (IFs) provide structural stability to the mammalian Golgi. Some experiments (BFA-wash-out, EM studies Golgi integrity in cells growing on high stiffness hydrogels) indeed support a functional connection between vimentin and Golgi ribbon integrity, but, on the other hand, Golgi alterations in vimentin KO cells could be an indirect consequence of previously published vimentin role in the morphological organization of ER. Data shown in the paper are insufficient to support the authorÂ’s idea that the endogenous GORAB is associated with vimentin IF. To ensure that the vimentin-Golgi connection is direct, authors should better characterize GORAB-vimentin interaction at the endogenous level and/or characterize Golgi protein(s) that are responsible for vimentin-Golgi interaction and demonstrate that alteration of specific vimentin-Golgi interactions results in Golgi defects.

Comments for the author

Specific points:
1. GORAB-BirA experiments are performed with overexpressed GORAB protein.
While this GORAB-BirA construct is mostly Golgi-localized (Figure S1), we do not know the extent of GORAB overexpression. GORAB WB will help here. This is important since the author’s data indicate that overexpressed GORAB shows significant co-localization with IFs (Figure S2).
2. There is no internal control for GORAB-BirA proteomics (like soluble BirA). Vimentin is often biotynilated in BioID experiments, and simple comparison with published data is insufficient for validation of GORAB-Vimentin interaction
3. GORAB-Vimentin interaction should be validated at the endogenous level.
The Abstract statement “the trans-Golgi network coiled-coil protein GORAB can physically associate with IFs” is only partially valid for the overexpressed GORAB.
4. Co-localization experiments (Figures 1 and S2) should be quantified. It has been mentioned that vimentin colocalizes with both Golgi markers GM130 and TGN46, but there is no unbiased colocalization analysis with those markers shown. In addition, vimentin seems aligned with GORAB but not with TGN46 in Figure 1C (graph).
5. The authors observed no major change in the morphology or positioning of the ER, ERGIC, or the Golgi apparatus in the GORAB or vimentin single and double knockout cells. This result contradicts the proposed role of vimentin in Golgi structural stability.
6. Golgi ribbon is also intact in KO and DKO cells which makes the statement doubtful that vimentin plays a role in the structural stability of Golgi.
7. Mammalian Golgi is disassembled/reassembled during cell division, and this assay is more relevant to cell physiology than the BFA wash-out assay.
Authors should test if Golgi re-assembly after mitosis is deficient in vimentin and GORAB KO cells.
8. It will be essential to check the status of ER and nuclear envelope in cells shown in Figure 6.

First revision

Author response to reviewers' comments

We have also included a separate file for the response to reviewers' comments as supplementary information. Please use this one since it contains formatting and an image that may be lost in this text box.

Reviewer 1 Advance Summary and Potential Significance to Field:
The manuscript by Vitali et al investigates the role of the intermediate filament vimentin on the

© 2023. Published by The Company of Biologists under the terms of the Creative Commons Attribution License (https://creativecommons.org/licenses/by/4.0/).

integrity of the mammalian Golgi ribbon structure. Their studies provide evidence that vimentin filaments are in close proximity to the Golgi resident protein GORAB. Loss of vimentin does not seemingly alter the Golgi ribbon under steady state conditions but affects the Golgi morphology in cells plated on matrices with increased stiffness. Vimentin depletion further altered the dynamics of disassembly and reassembly of the Golgi after brefeldin A treatment. Based on the results, the authors conclude that vimentin stabilizes the Golgi structure.
Whether intermediate filaments mechanically support intracellular organelles such as the Golgi has been an unresolved question. This study directly shows that vimentin provides stability to the integrity of the Golgi ribbon morphology and as such makes an important contribution to the field. The data are clear and of very high quality. The manuscript is well written and should be published in JCS.

Reviewer 1 Comments for the Author:
There are only few minor suggestions the authors could consider:

1. The authors show that the MT network appears to be normal under steady-state conditions in vimentin ko cells, but it is not clear if MT dynamics are changed. The Golgi structure and integrity depends on the MT network and MTs physically interact with vimentin filaments to stabilize dynamic MT polymers (PMID: 34145230). As such it is possible that a changed MT turnover affects Golgi stability. The authors could discuss/test the possibility that Golgi ribbon disassembly and reformation dynamics are changed when MTs are depolymerized and reform. Similarly, the authors could stain for MTs in Fig.6. The MT network in vimentin ko cells might be altered depending on the stiffness of the culture matrix, which in turn could affect Golgi integrity.
Response: We have performed the suggested experiment using nocodazole to depolymerise microtubules and fragment the Golgi apparatus, as well as a washout of nocodazole to allow Golgi reassembly. We observed a more rapid disassembly of the Golgi treatment in vimentin-null cells upon nocodazole treatment, and a slower reassembly upon nocodazole washout. Thus, we still see changes in Golgi stability in the absence of vimentin when microtubule dynamics are altered, supporting the view that vimentin is acting independently of microtubules in this role. The data are included in a new Fig S6. In further support of this conclusion, we also labelled the microtubules in cells grown on different stiffness hydrogels, as suggested. We did not see any major changes in microtubule organisation upon loss of vimentin under these conditions, which is included in Fig S5C.

2. In Fig.3 the authors use EM to evaluate the ultrastructure of Golgi stacks. The number of cisternae per stack is significantly reduced and the length of the cisternae are shorter. Although this is a quite a big change, the author conclude that "GORAB and vimentin do not play a major role in Golgi organization, polarity or positioning under steady state conditions." Please explain the reasons for this conclusion. By looking at the EM images it also appears to me that the number of vesicles in the Golgi area is substantially increased. Loss of cisternae, shortened cisternae and more vesicles (and the changes in dynamics induced by brefeldin A) may also point to a membrane transport defect. The authors should discuss this possibility.
Response: We have noted the increase in spherical profiles in the EM of the knockout cells on page 7 of the text. We also discuss the possibility of a Golgi trafficking defect in the Discussion section (page 11).

3. Vimentin knock out mice are normal and do not show an obvious phenotype (PMID: 7954832). Does this mean that the changes in Golgi structure in vimentin depleted cells are irrelevant to the animals?
Response: This initial study on the generation of a vimentin knockout mouse has been followed by numerous studies reporting a range of phenotypes in these animals, which is nicely summarised in a recent review by Robert Goldman and colleagues (PMID: 35487686). It is difficult to know the extent to which Golgi disruption may contribute to the various phenotypes reported, but it certainly plausible that Golgi disruption may play a role a role in at least some of these phenotypes. We discuss this on page 11 of the Discussion.

4. Overexpressed GORAB forms filament-like structures which are dependent on vimentin. The

© 2023. Published by The Company of Biologists under the terms of the Creative Commons Attribution License (https://creativecommons.org/licenses/by/4.0/).

authors conclude that these GORAB filaments assemble on preexisting vimentin IF, which they argue is consistent with a “physical interaction” (page 5 and abstract). The fact that overexpressed GORASP forms filaments that colocalize with vimentin does not necessarily mean that the two proteins physically interact. Similarly, on page 10 it is discussed that GORAB oligomerization is required for vimentin binding. The experiments presented in this manuscript do provide evidence for binding. Fig.S2 merely shows colocalization of overexpressed GORAB with vimentin filaments, but not binding. I suggest changing the text from “binding” to “colocalization”.
Response: We agree with the reviewer’s comments and tried to be cautious in our description of the data in the original version of the manuscript. To provide additional support for an association between GORAB and vimentin in cells, we used a proximity ligation approach. This indicated interaction, or close physical association, between the proteins. Various controls including the use of GORAB null cells confirmed the specificity of the interaction. This data is included in a new Fig 1G and H. In light of this new data we prefer to keep the text describing a physical association between the proteins.

Reviewer 2 Advance Summary and Potential Significance to Field:
In this manuscript, Vitali, Lowe and coll. question the role of vimentin and intermediate filaments in Golgi organization in particular through the function of the GORAB protein.

Altogether, this is an interesting study that explores an overlooked role played by microfilaments on the control of Golgi dynamics and localization. Results are in general convincing and well analyzed, and the reported observations would open novel questions in membrane cell biology. In conclusion, I think that this study may be published by the Journal of Cell Science after considering the points raised bellow.

Reviewer 2 Comments for the Author:
1. The main question raised by the approach is whether over-expression of GORAB, that modifies strongly its localization, is meaningful. I think it would help a lot to add a few complementation experiments like a rescue of GORAB KO effects (on Golgi organization?) with a K190 deletion mutant or an immunoprecipitation done at endogenous expression levels.
Response: To address this point, we used a proximity ligation approach to assess interaction between GORAB and vimentin, using antibodies to the endogenous proteins. The experiments confirmed binding, or at the least very close physical proximity, between the proteins, when present at endogenous levels. Various controls, including the use of GORAB- null cells, confirmed the specificity of the result. This data is included in a new Fig 1G and H. It is also worth noting that the Bio-ID experiment was performed with cells expressing low levels of BirA-tagged GORAB. Western blotting with anti-GORAB antibodies indicated only a 2-fold increase in expression over endogenous GORAB. We have now included this Western blotting data in Fig S1B of the revised manuscript, and commented in the text on the modest level of over-expression.

2. The second main difficulty I have with this study is the impact of substrate stiffness on Golgi organization. The authors are aware of this and discuss it, but I am still puzzled. On the one hand, no effect is seen on a hyper-stiff glass substrate while it is clearly observed when cultivating cells on 100 kPa substrate. For dKO cells, effect is actually seen on all rigidity (to different levels) but not on glass. The authors may like to stain microtubules/actin of cells seeded on substrates with different stiffness and glass to try to understand better what is happening.
To me, this is not central to the study and because the authors have no experiments to explain this different behavior, I am wondering whether the authors should not choose to remove these data and invest on a larger set of experiments to understand this in a later study.
Response: We agree that the glass versus hydrogel stiffness data seem paradoxical. To try to understand this better we labelled microtubules and actin of cells grown on different stiffness hydrogels, as suggested. The data did not show any major difference in the organisation of these cytoskeletal elements under the different conditions, so we think any changes in structural integrity of the Golgi on different stiffness hydrogels are independent of these elements. The new data is shown in Fig S5C and D. It is known that substrate chemistry, in combination with stiffness, is a key factor in cell behaviour when growing in different stiffness environments, which makes it difficult to directly compare the results on glass versus hydrogels. However, we believe the

© 2023. Published by The Company of Biologists under the terms of the Creative Commons Attribution License (https://creativecommons.org/licenses/by/4.0/).

hydrogel data is robust, and because the only variable in these experiments is stiffness, we are correct in concluding that vimentin is important for Golgi integrity in stiffer versus less stiff environments. Because the experiments are in vitro, it is difficult to know how this would translate to the in vivo situation, but we hypothesise that the Golgi will be more dependent on vimentin for its integrity in stiffer tissues or those under more mechanical stress compared to the softer tissues.

3. The main effect reported upon Vimentin deletion is on Golgi disassembly/reassembly. While it may intuitively be simple to imagine why a loss of a Vimentin scaffold would allow for faster disassembly, it is harder to imagine why reassembly would be slowed down in these conditions. I think it would be important to complement these data with experiment (1) monitoring in real-time Golgi disassembly and (2) tracking the behavior of Golgi proteins that are, in contrast to GM130, quantitatively transported to the ER upon BFA treatment (like a GFP-tagged Golgi enzyme [or simply its trans-membrane domain as often done]). The presence of tubules, or not, at early stages after BFA addition would be interesting to analyze as well.
Response: We have performed live imaging of cells expressing NAGFP, a chimera between the stack and transmembrane regions of the Golgi enzyme NAGTI and GFP (PMID: 9182657). This confirmed the more rapid loss of signal from the Golgi region in vimentin KO cells, together with more rapid and extensive appearance of NAGFP-containing tubules in the KO cells compared to control. The increased tubulation is consistent with reduced structural integrity of the Golgi upon loss of vimentin. We also performed live imaging of BFA washout in the NAGFP-expressing cells. The live imaging data are included in Fig S3C-E (Movies 1-4) and Fig S7B (Movies 5-6).

4. Still in the frame of the “scaffolding” model proposed by the authors, addition a set of nocodazole/nocodazole washout experiment would be interesting to add as well.
Intriguingly, data shown in Figure 3 suggest that Vimentin KO impacts localization of GORAB on mini stacks and the extend of dispersion in the cytosol but this would need quantification, and, as suggested above, real-time experiments.
Response: A similar suggestion was made by reviewer 1. We performed the experiment using nocodazole to depolymerise microtubules and fragment the Golgi apparatus, as well as a washout of nocodazole to allow Golgi reassembly. We observed a more rapid disassembly of the Golgi treatment in vimentin-null cells upon nocodazole treatment, and a slower reassembly upon nocodazole washout. Thus, we see changes in Golgi stability using another means to induce disassembly and reassembly of the organelle. The new data are included in Fig S6.

5. The authors indicate that GORAB can physically associate with IF but to me no such data is presented. As already proposed above, direct interaction may be looked for by the authors, in particular between endogenous (and not overexpressed) GORAB and Vimentin.
Response: To provide additional evidence that GORAB and vimentin interact, we performed proximity ligation. In this experiment, we used antibodies to the endogenous proteins, and could detect interaction (or at least very close physical association) in wild-type cells, but not in GO patient cells devoid of GORAB. These data strengthen our conclusion that the proteins interact in cells. The data are included in a new Fig 1G and H.

6. As a more minor remark, it is quite strange to enter so deeply in the study with many supplementary figures before referring to main figures. I would propose to move Fig S1 as a main figure as it is an important starting point.
Also, in this Figure the authors need to homogenize bioID/BirA nomenclature.
Response: We agree that referring only to supplementary figures for the first part of the paper is not ideal. We decided to keep the characterisation of the GORAB-BirA cell lines as a supplementary figure, but moved the data showing colocalisation of over-expressed GORAB with vimentin into a main figure (Fig 1). The new PLA data was also added the to this figure. The BirA/BIOID nomenclature has also been homogenized in Fig S1.

7. Fig 2A : there seem to be a lower expression of GORAB in Vimentin KO cells. Is this significant?
Response: We have analysed GORAB expression in the vimentin KO cells in numerous experiments and do not see any significant change in its abundance compared to controls, using both Western blotting and IF microscopy. We include here an additional blot showing GORAB levels in the vimentin KO MEFs and a second line where we knocked out vimentin using CRISPR-Cas9. The levels

© 2023. Published by The Company of Biologists under the terms of the Creative Commons Attribution License (https://creativecommons.org/licenses/by/4.0/).

of GORAB are unaffected by vimentin KO.

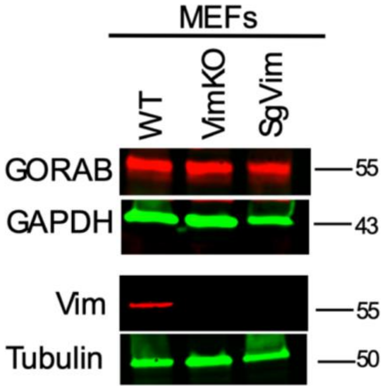

8. Quantification of Golgi elements was done by counting the number of elements above the intensity threshold of the Golgi in MEFwt cells, is total intensity of the Golgi marker the same in the different conditions? (Figure 2 suggests a lower expression that may impact image analysis)
Response: In the original Figure 6 (now Fig 7; hydrogel data) and the new microtubule disassembly/reassembly data (Fig S6), we used a number of Golgi markers to quantify Golgi elements. The abundance of the markers (GM130, GORAB, Stx6) is not different in the KO cells, as judged by Western blot (GM130) and immunofluorescence intensity (GM130, GORAB, Stx6) in untreated cells. We are therefore confident that the quantification of Golgi elements is not confounded by reduced abundance of the markers used.

9. There is a “funny” typo in legend of figure S2 “residual lowe level expression”
Also, “olgomerization” is indicated in the second page of the discussion
Response: The spelling mistakes have been corrected.

Reviewer 3 Advance Summary and Potential Significance to Field:
In the manuscript by Vitali et al, the authors described a set of interesting and, for the most part, well-controlled experiments in support of their hypothesis that vimentin intermediate filaments (IFs) provide structural stability to the mammalian Golgi. Some experiments (BFA-wash-out, EM studies, Golgi integrity in cells growing on high stiffness hydrogels) indeed support a functional connection between vimentin and Golgi ribbon integrity, but, on the other hand, Golgi alterations in vimentin KO cells could be an indirect consequence of previously published vimentin role in the morphological organization of ER. Data shown in the paper are insufficient to support the author’s idea that the endogenous GORAB is associated with vimentin IF. To ensure that the vimentin-Golgi connection is direct, authors should better characterize GORAB-vimentin interaction at the endogenous level and/or characterize Golgi protein(s) that are responsible for vimentin-Golgi interaction and demonstrate that alteration of specific vimentin-Golgi interactions results in Golgi defects.

Reviewer 3 Comments for the Author:
Specific points:
1. GORAB-BirA experiments are performed with overexpressed GORAB protein. While this GORAB-BirA construct is mostly Golgi-localized (Figure S1), we do not know the extent of GORAB overexpression. GORAB WB will help here. This is important since the author’s data indicate that overexpressed GORAB shows significant co-localization with IFs (Figure S2).
Response: As suggested we have performed Western blotting of the HeLa cells stably expressing GORAB-BirA that were used for the mass spectrometry analysis with antibodies to GORAB. This indicates a modest overexpression of the BirA-tagged GORAB (2-fold). This new data is shown in Fig S1B.

2. There is no internal control for GORAB-BirA proteomics (like soluble BirA). Vimentin is often biotynilated in BioID experiments, and simple comparison with published data is insufficient for validation of GORAB-Vimentin interaction
Response: We did not use soluble BirA for the proteomics. We agree this would have been a

© 2023. Published by The Company of Biologists under the terms of the Creative Commons Attribution License (https://creativecommons.org/licenses/by/4.0/).

valuable control, but were also worried that if GORAB did bind cytosolic proteins, then we may have excluded them from our subsequent analysis. Hence, we resorted to comparison with existing datasets, but we also acknowledge this is not ideal either. To obtain more evidence for a physical association between GORAB and vimentin at endogenous levels we used proximity ligation. This indicated interaction, or at least close physical proximity, between the proteins. Various controls including the use of GORAB null cells confirmed the specificity of this interaction. This new data is included in Fig 1G and H.

3. GORAB-Vimentin interaction should be validated at the endogenous level. The Abstract statement “the trans-Golgi network coiled-coil protein GORAB can physically associate with IFs” is only partially valid for the overexpressed GORAB.
Response: To address this point we used proximity ligation, with antibodies to GORAB and vimentin. This indicated an interaction, or at least very close physical proximity, between the endogenous proteins. GOARB null cells and antibody controls confirmed the specificity of this result. These data support our conclusion that GORAB and vimentin associate at endogenous levels, and are included in a new Fig 1G and H.

4. Co-localization experiments (Figures 1 and S2) should be quantified. It has been mentioned that vimentin colocalizes with both Golgi markers GM130 and TGN46, but there is no unbiased colocalization analysis with those markers shown. In addition, vimentin seems aligned with GORAB but not with TGN46 in Figure 1C (graph).
Response: It is extremely challenging to accurately quantify the extent of colocalisation of the Golgi markers within the meshwork of vimentin filaments that are concentrated in the perinuclear region of the cell. Using Pearson’s or Mander’s correlation or overlap coefficients will give a high spurious reading due to concident overlap in this region of the cell. We therefore believe the best way to show the spatial relationship between the two structures is through the zooms and linsecans we have used. We acknoweldge this is limited, and have tried to describe this fairly in the text, where we refer to a close spatial relationship between the structures, with frequent co-alignment, as opposed to solely a colocalisation. In the original Fig 1C (now Fig 2C), the top linescan shows TGN46 closely co- aligned with two of the vimentin peaks.

5. The authors observed no major change in the morphology or positioning of the ER, ERGIC, or the Golgi apparatus in the GORAB or vimentin single and double knockout cells. This result contradicts the proposed role of vimentin in Golgi structural stability.
Response: Our interpretation is that the Golgi integrity is reduced in vimentin-null cells, but not to the extent where cells growing at steady state on glass show a major change in Golgi morphology. However, a phenotype does manifest on stiff hydrogels, and we see changes in rates of Golgi disassembly upon chemical disruption with BFA or nocodazole. Thus, we think the Golgi is more ‘fragile’ in the absence of vimentin, resulting in the phenotypes observed. We need not expect changes in ER or ERGIC organisation to accompany the changes we see in Golgi morphology upon loss of vimentin. They are clearly functionally linked but the Golgi ribbon is a different physical entity to the ERGIC and ER.

6. Golgi ribbon is also intact in KO and DKO cells which makes the statement doubtful that vimentin plays a role in the structural stability of Golgi.
Response: Please see our response to point 5 above.

7. Mammalian Golgi is disassembled/reassembled during cell division, and this assay is more relevant to cell physiology than the BFA wash-out assay. Authors should test if Golgi re- assembly after mitosis is deficient in vimentin and GORAB KO cells.
Response: This would be a very interesting experiment. However, it is challenging to perform, and may also be confounded by recent studies showing a role for vimentin in mitosis (PMID: 31519880; PMID: 31928973). We have used nocodazole as another way to induce Golgi disassembly and its washout to assess reassembly. Our results indicate faster disassembly and slower reassembly in the vimentin KO cells compared to control in these experiments, which is included in a new Fig S6.

8. It will be essential to check the status of ER and nuclear envelope in cells shown in Figure 6.
Response: We performed immunofluorescence microscopy on cells growing on different stiffness

© 2023. Published by The Company of Biologists under the terms of the Creative Commons Attribution License (https://creativecommons.org/licenses/by/4.0/).

hydrogels with antibodies to PDI and lamin B, to assess endoplasmic reticulum and nuclear morphology respectively. We did not observe significant changes in the shape or size of either organelle on the different stiffness substrates. We did not perform a detailed quantitative analysis, so we cannot exclude the possibility of minor changes in the morphology of these organelles. Although it has been reported previously that the nucleus changes shape in response to different stiffness environments, the magnitude of the changes is rather modest e.g. PMID 23914256. We would not expect to see such minor changes at a gross level, especially also considering ER and nucleus size changes as cells grow, and we did not synchronize the cell cycle in these experiments. The new data are included in Fig S5C and D.

Second decision letter

MS ID#: JOCES/2022/260577

MS TITLE: Vimentin intermediate filaments provide structural stability to the mammalian Golgi apparatus

AUTHORS: Teresa Vitali, Rosa Sanchez-Alvarez, Tomasz M Witkos, Ioannis Bantounas, Marie F Cutiongco, Michal Dudek, Guanhua Yan, Alexander A Mironov, Joe Swift, and Martin Lowe
ARTICLE TYPE: Research Article

I am happy to tell you that your manuscript has been accepted for publication in Journal of Cell Science, pending standard ethics checks.

© 2023. Published by The Company of Biologists under the terms of the Creative Commons Attribution License (https://creativecommons.org/licenses/by/4.0/).
